# spliceJAC: transition genes and state‐specific gene regulation from single‐cell transcriptome data

**DOI:** 10.15252/msb.202211176

**Published:** 2022-11-02

**Authors:** Federico Bocci, Peijie Zhou, Qing Nie

**Affiliations:** ^1^ Department of Mathematics University of California Irvine CA USA; ^2^ NSF‐Simons Center for Multiscale Cell Fate Research University of California Irvine CA USA; ^3^ Department of Developmental and Cell Biology University of California Irvine CA USA

**Keywords:** attractor linear stability, cell state transition, gene regulatory network, mRNA splicing, single‐cell RNA sequencing, Computational Biology, Methods & Resources

## Abstract

Extracting dynamical information from single‐cell transcriptomics is a novel task with the promise to advance our understanding of cell state transition and interactions between genes. Yet, theory‐oriented, bottom‐up approaches that consider differences among cell states are largely lacking. Here, we present spliceJAC, a method to quantify the multivariate mRNA splicing from single‐cell RNA sequencing (scRNA‐seq). spliceJAC utilizes the unspliced and spliced mRNA count matrices to constructs cell state‐specific gene–gene regulatory interactions and applies stability analysis to predict putative driver genes critical to the transitions between cell states. By applying spliceJAC to biological systems including pancreas endothelium development and epithelial–mesenchymal transition (EMT) in A549 lung cancer cells, we predict genes that serve specific signaling roles in different cell states, recover important differentially expressed genes in agreement with pre‐existing analysis, and predict new transition genes that are either exclusive or shared between different cell state transitions.

## Graphical abstract

spliceJAC builds a multivariate mRNA splicing model from single‐cell transcriptome data to infer the context‐specific gene regulation and the key driver genes that guide the transition between cell states.

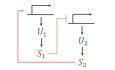

## Introduction

The recent explosion of single‐cell transcriptomics methods has provided unprecedented resolution on studying single‐cell processes (Aldridge & Teichmann, 2020), allowing one to closely inspect the dynamics of cell‐fate decisions (MacLean *et al*, 2018). To dissect the state‐transition dynamics from single‐cell snapshot data(Weinreb *et al*, 2018), identify key genes that drive the transitions between cell types (Chen *et al*, 2019), and infer cell type‐specific gene regulations (Wang *et al*, 2020), the need of the bottom‐up, theory‐oriented, and interpretable frameworks to analyze the dynamical systems of cell state transitions (Moris *et al*, 2016; preprint: Xing, 2022) is becoming increasingly clear. Despite some recent progresses (Bargaje *et al*, 2017; Zhou *et al*, 2021; Qiu *et al*, 2022), theoretical and computational analysis beyond the purely statistical or machine learning approaches (Luecken & Theis, 2019) remains largely unexplored.

The possibility to distinguish intronic and mature mRNAs in single‐cell RNA sequencing (scRNA‐seq) has improved our ability to infer dynamical information from static scRNA‐seq snapshots (Tritschler *et al*, 2019; Li *et al*, 2021; Gorin *et al*, 2022). For instance, the RNA velocity framework models the splicing dynamics of nascent mRNAs to predict future gene expression patterns and transitions (La Manno *et al*, 2018; Bergen *et al*, 2020). This approach can recapitulate differentiation trajectories in developmental systems such as dentate gyrus neurogenesis and pancreatic endocrinogenesis (La Manno *et al*, 2018; Bergen *et al*, 2020). One major limitation of this approach is that different cell populations often exhibit distinct kinetic regimes (preprint: Cui *et al*, 2022). An interesting example is the dentate gyrus neurogenesis, where an endothelial cell population exhibits a distinct slope in the spliced/unspliced space (Bergen *et al*, 2021). Another clinically relevant scenario is heterogeneous tumors, where cells can undergo reversible transitions along different axes including epithelial–mesenchymal transition (EMT), acquisition of cancer stem cell (CSC) traits, metabolic reprogramming and leader‐follower during collective migration (Shibue & Weinberg, 2017; Jia *et al*, 2019; Faubert *et al*, 2020; Mercedes *et al*, 2021), thus exhibiting multiple coexisting cell states corresponding to different phenotypes. Moreover, current models consider the splicing of different mRNA species within the same cell as independent processes, thus disregarding transcriptional regulation (Bergen *et al*, 2021). Integrating transcriptional regulation can not only refine current models of mRNA splicing but also provide a new avenue to learn feedback regulations between genes.

Currently, several “global” methods aim at reconstructing emergent gene–gene interaction networks using conventional (i.e., only spliced) mRNA count data (Huynh‐Thu *et al*, 2010; Kim, 2015; Chan *et al*, 2017; Matsumoto *et al*, 2017; Specht & Li, 2017; Gao *et al*, 2018; Sanchez‐Castillo *et al*, 2018; Woodhouse *et al*, 2018; Moerman *et al*, 2019; Aubin‐Frankowski & Vert, 2020; Deshpande *et al*, 2022). A potential drawback of such an approach is that, arguably, many key interactions are context‐specific and therefore present only in certain cell states. Moreover, since snapshot single‐cell datasets lack sufficient dynamical information for the comprehensive inference (Wang & Wang, 2022), many existing GRN inference methods rely on pseudotime ordering of cells to create “trajectories,” which itself require additional assumptions and may lead to significant problems in performance when compared with true time series data (Qiu *et al*, 2020).

To tackle these limitations, we present spliceJAC, a multivariate analysis of mRNA splicing that account for multiple coexisting cell states and their state‐specific gene regulation. Following a parallel with the Waddington landscape (Waddington, 1957; Wang *et al*, 2011; Zhou *et al*, 2012; Zhou & Li, 2016) methodology, we interpret cell states as attractors separated by barriers in an underlying high‐dimensional landscape (Huang *et al*, 2005). Cells within each attractor are characterized by a unique set of gene–gene interactions that define their specific gene expression pattern and “unstable” genes capable to drive transitions toward new cell states. One major advantage of spliceJAC is that the predicted gene–gene interaction matrices also encode information about the stability of individual cell states. More specifically, spliceJAC characterizes each cell state with a Jacobian matrix, whose spectral analysis provides key information about the most unstable genes that potentially drive transitions toward other cell states.

In this work, we first present a theoretical framework for the multivariate mRNA splicing. Next, we introduce spliceJAC, an easy‐to‐use python package that implements the model and offers several options for downstream analysis. Furthermore, we test spliceJAC on several *in silico*, multistable circuits, where we show it outperforms several existing methods for GRN inference from scRNA‐seq data. Moreover, we analyze two systems, including mouse pancreas endothelium development and epithelial–mesenchymal transition of A549 lung carcinoma cells, where spliceJAC uncovers context‐specific gene regulation and predicts new transition genes not identified with existing methods based on gene expression.

## Results

### Modeling stability and cell state‐specific regulation with multivariate mRNA splicing analysis

The spliceJAC is motivated by nonlinear dynamical system modeling of gene expression processes in single cells. The interconnections between genes within a cell can be modeled with a system of nonlinear ordinary differential equations:

(1) $$\dot{x}=F\left(x\right)$$

where $x=\left(x_{1},x_{2},\ldots ,x_{N}\right)$ is the vector of protein counts, $\dot{x}$ is its time derivative, and $F\left(x\right)$ is a generic, nonlinear force field that models interactions between proteins as well as any other cellular process of interest. A common explicit form for the force field acting on molecular species ($x_{i}$) is as follows:

(2) $$\frac{dx_{i}}{\mathrm{dt}}=k_{i}f^{i}\left(x_{1},x_{2},\ldots ,x_{N}\right)-\gamma_{i}x_{i}$$

where $k_{i}$ and $\gamma_{i}$ are basal production and degradations rate constants, and $f^{i}\left(x_{1},x_{2},\ldots ,x_{N}\right)$ is a nonlinear function of all chemical species acting on species i. This model can be generalized to include mRNA splicing dynamics (La Manno *et al*, 2018; Bergen *et al*, 2020), that is, the intermediate step where intronic, noncoding RNA regions are removed from newly produced mRNA transcripts:

(3a) $$\frac{dU_{i}}{\mathrm{dt}}=k_{i}f^{i}\left(S_{1},S_{2},\ldots ,S_{N}\right)-\beta U_{i}$$

(3b) $$\frac{dS_{i}}{\mathrm{dt}}=\beta U_{i}-\gamma_{i}S_{i}$$

where $U_{i}$ and $S_{i}$ are copy numbers for unspliced and spliced mRNA of species i, and $f^{i}\left(S_{1},S_{2},\ldots ,S_{N}\right)$ is a nonlinear function that represents the regulatory effect from all molecular species quantified by the mature mRNAs. Finally, β is a splicing rate constant that is assumed to be equal for all mRNA species. Therefore, equations (3a) and (3b) can be interpreted as a generalization of existing mRNA splicing models (La Manno *et al*, 2018; Bergen *et al*, 2020) that includes multivariate mRNA dynamics (Bergen *et al*, 2021). Equations (3a) and (3b) can generally exhibit one or more attractors that are associated with distinct cell states with different gene expression profiles. When close enough to an attractor, gene regulation functions can be linearized:

(4) $$f^{i}\left(S_{1},S_{2},\ldots ,S_{N}\right)\approx \begin{array}{l} f^{i}\left({S_{1}}^{\mathrm{ss}},{S_{2}}^{\mathrm{ss}},\ldots ,{S_{N}}^{\mathrm{ss}}\right)+\sum_{j=1}^{N}A_{\mathrm{ij}}\left(S_{j}-{S_{j}}^{\mathrm{ss}}\right)\\ +O\left({\left(S_{j}-{S_{j}}^{\mathrm{ss}}\right)}^{2}\right) \end{array}$$

where superscript *ss* implies “steady state.” Therefore, nearby each attractor of equations (3a) and (3b), a linear approximation based on equation (4) simplify the nonlinear regulation to a summation of linear terms:

(5a) $$\frac{dU_{i}}{\mathrm{dt}}=A_{i0}+\sum_{j=1}^{N}A_{\mathrm{ij}}S_{j}-\beta U_{i}$$

(5b) $$\frac{dS_{i}}{\mathrm{dt}}=\beta U_{i}-\gamma_{i}S_{i}$$

where $A_{i0}=f^{i}\left({S_{1}}^{\mathrm{ss}},{S_{2}}^{\mathrm{ss}},\ldots ,{S_{N}}^{\mathrm{ss}}\right)$ is a basal production rate, and the coefficients $A_{\mathrm{ij}}$ describe the regulation of any given molecular species $S_{j}$ on $U_{i}$. While equation (5b) is similar to existing RNA velocity models, equation (5a) includes local (i.e., cell state‐specific) gene–gene regulation. Therefore, while equations (3a) and (3b) can describe a nonlinear regulatory circuit with potentially multiple stable states, we linearize the circuit interactions around each stable cell states that are thus described by different parameters. Assuming steady state near each fixed point (i.e., $\frac{dU_{i}}{\mathrm{dt}}=0$), equation (5a) allows to infer an attractor‐specific gene–gene interaction matrix (Appendix Methods, section 1). To ensure a unique solution to the regression problem, the number of genes cannot exceed the number of observations (i.e., cells). Therefore, the size of the number of cells within a given cell state defines as an upper limit to the number of inferred interactions. It is worth noting that equation (5a) and (5b) remain valid even when the multivariate term $f^{i}\left(S_{1},S_{2},\ldots ,S_{N}\right)$ is a product of individual regulatory functions, an often‐used modeling strategy in systems biology (Appendix Methods, section 1). The following section provides an overview of spliceJAC, a package that implements this formalism to infer cell type‐specific regulatory interactions and predict critical transition driver genes.

### Overview of spliceJAC

**Figure 1:**
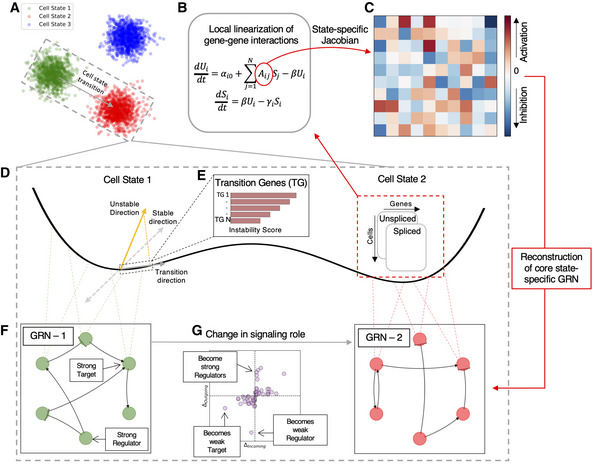
Overview of spliceJAC and main analysis output As input, spliceJAC requires unspliced and spliced mRNA count matrices as well as cell annotations.spliceJAC fits the mRNA count data within each cell state to a multivariate mRNA splicing model.The output of the model is a set of gene–gene interaction matrices that encode gene–gene interactions in each cell state.The switch between two cell states is interpreted as a transition on a high‐dimensional landscape shaped by the underlying state‐specific gene regulatory networks.By projecting the unstable eigenvalues of the inferred Jacobian matrix, spliceJAC predicts key transition genes (TG).Each cell state exhibits specific interactions between genes that are captured in a regulatory network. Downstream analysis of the network identifies important signaling hubs such as strong regulators that modulate many other genes and strong targets that receive inputs from multiple genes.A state transition coincides with a rearrangement of the state‐specific regulatory network.

spliceJAC requires mRNA count matrices of unspliced and spliced mRNA and cell annotations as user input (Fig 1A). In the spliceJAC framework, it is assumed that cell annotations (i.e., cluster labels) correspond to stable cell states. This, however, is not necessarily true, and deciding which cell subpopulations can be effectively considered as stable states depends on the specific propertied and prior knowledge on the considered biological system. Downstream stability analysis of spliceJAC (described below) can provide the self‐consistent validation for such assumption.

In the inference step, spliceJAC computes cell state‐specific gene–gene interaction matrices (or, more precisely, Jacobian matrices) by modeling the coupled mRNA splicing dynamics (Fig 1B and C, Methods and Protocols: Inference of cell state specific gene‐gene interactions from multivariate mRNA splicing analysis). Upon gene–gene interaction inference, spliceJAC performs downstream analysis based on dynamical system theory, focusing on three areas: (i) individual cell states; (ii) transitions between cell states; and (iii) comparison between transitions stemming from a shared starting state.

#### Identifying individual cell states

First, at the level of individual cell states, spliceJAC computes a core regulatory network with state‐specific gene–gene interactions (Fig 1F). The number of selected key interactions and size of the network can be controlled by the user based on a quantile selection feature. From the gene–gene interaction information, signaling scores can be computed based on the incoming and outgoing interaction stemming from each node, which can be visualized in a two‐dimensional scatterplot to identify the key signaling hubs of each cell type (Methods and Protocols: Quantification of signaling roles and signaling change upon transition). Moreover, a global comparison between cell types identifies genes with context‐specific or conserved functions across cell types (Methods and Protocols: Signaling variability of individual genes across cell states and Top differential/conserved interactions and GRN comparison between cell states). Finally, from the gene–gene interaction matrix, it is possible to evaluate the stability of the cell state and potential unstable directions (Methods and Protocols: Identification of transition driver genes from Jacobian eigen‐space analysis).

#### Analysis of transitions between cell states

Second, spliceJAC provides information about the transitions between cell states. A set of transitions of interest can be provided as a user input. To identify important transition driver genes, spliceJAC projects the starting state's unstable manifold onto the path connecting starting and target cell states, thus considering the specific transition direction in the gene landscape (Fig 1D and E and Methods and Protocols: Identification of transition driver genes from Jacobian eigen‐space analysis). The propensity of genes to drive the transition is quantified by an “instability score” bound between 0 (very stable, not inducing the transition) and 1 (very unstable, drives the transition) (Fig 1E). To elucidate how transition driving genes connect to key, highly expressed genes, spliceJAC features a dedicated plotting function that highlights a core GRN including the top differentially expressed genes (DEGs) of the starting cell state and the top transition genes leading to the final cell state. Finally, comparing gene–gene interactions between starting and final states provides information about the genes with significant changes as regulators or targets upon transition, which can be visualized as two‐dimensional scatter plots (Fig 1G) or as a GRN of differential interactions.

#### Comparison of transitions from the same starting state

In biological scenarios where cells can access one of multiple, distinct states, spliceJAC compares the individual transition paths to identify driver genes that are specific to a single transition or shared between multiple transitions, respectively. Moreover, spliceJAC applies multiple metrics such as incoming signaling, outgoing signaling, and betweenness centrality (Methods and Protocols: Quantification of signaling roles and signaling change upon transition) to identify specific genes with standout signaling roles in only one, some, or all the final cell states.

### spliceJAC reconstructs state‐specific gene–gene interactions from *in silico* circuits

To test spliceJAC's ability to capture cell state‐specific gene–gene interactions, we evaluate its performance on *in silico* circuits where a ground truth can be computed analytically. To generate the *in silico* data, we consider small perturbations around each fixed point and the relaxation to the fixed point thereafter (Methods and Protocols: Simulation of *in silico* circuits).

**Figure 2:**
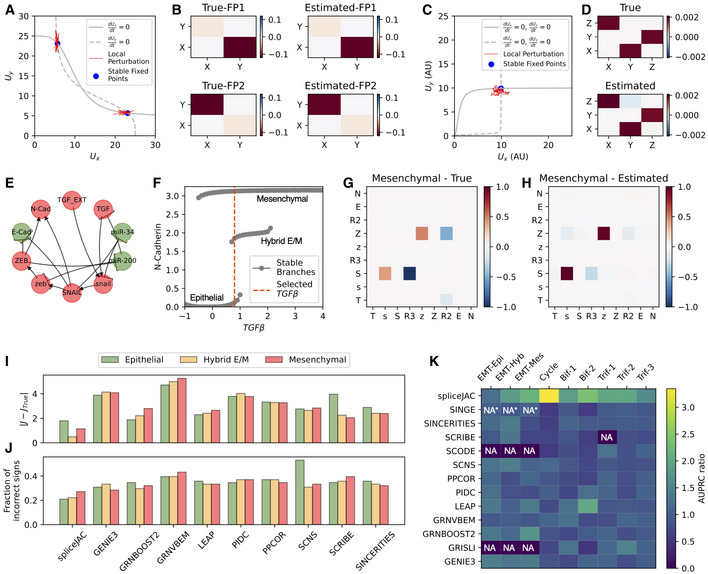
Benchmarking spliceJAC against *in silico* circuits and comparison with other existing GRN inference methods A
The phase space of a bistable toggle switch including nullclines (silver lines), stable fixed points (blue dots), and stochastic perturbation around stable fixed points (red lines). X‐ and y‐coordinates represent unspliced mRNA counts of genes X and Y.B
Ground truth (top) and inferred (bottom) interaction matrices for the two stable fixed.C
The phase space of a monostable circuit of three genes that activate each other in a loop.D
Ground truth and inferred interaction matrix of the three genes circuit.E
The EMT circuit proposed by Tian *et al* (2013). Green and red nodes highlight epithelial and mesenchymal genes, while pointing and t‐shaped arrows represent activation or inhibition, respectively.F
Bifurcation diagram showing the available attractors as a function of TGF‐beta inducer. The red dotted line highlights a value leading to tristability used for spliceJAC testing thereafter.G, H
Ground truth (G) and estimated (H) interaction matrices in the mesenchymal state.I, J
Comparison with existing GRN inference methods based on the absolute difference between ground truth and prediction (I) and maximization of the fraction of correct matrix element signs (J). Green, orange, and red bars showcase results for the Epithelia, Hybrid E/M and mesenchymal states, respectively.K
The AUPRC ratio for all methods in the Beeline pipeline and spliceJAC. For each circuit state, the AUPRC scores are normalized by the average score achieved for the state. NA = no output file was generated. NA* = an output file without any predicted edge was generated.

First, we consider a bistable toggle switch, an often‐used motif to simulate differentiation processes (Verd *et al*, 2014; Xu *et al*, 2014), composed by two genes (X, Y) that mutually inhibit each other (Appendix Methods, section 3), resulting in two symmetric fixed points: (high X, low Y) and (low X, high Y), respectively (Fig 2A). While both ground truth interaction matrices feature negative off‐diagonal elements corresponding to the mutual inhibition between X and Y, the amplitude of these interactions is reversed in the two states. In other words, only the X‐to‐Y inhibition arrow of the circuit is strongly activated in the (high X, low Y) state, and *vice versa*, thus underscoring the difference between the global network architecture and the amplitude of state‐specific interactions. spliceJAC reconstructs the interaction matrices from the simulated data with high degree of precision for both stable fixed points (Fig 2B). Furthermore, we consider a three‐gene loop with mutual activation that exhibits a single stable fixed point (Fig 2C; Appendix Methods, section 3). Similarly, spliceJAC correctly recovers the interaction matrix following the same simulation scheme (Fig 2D).

**Figure EV1:**
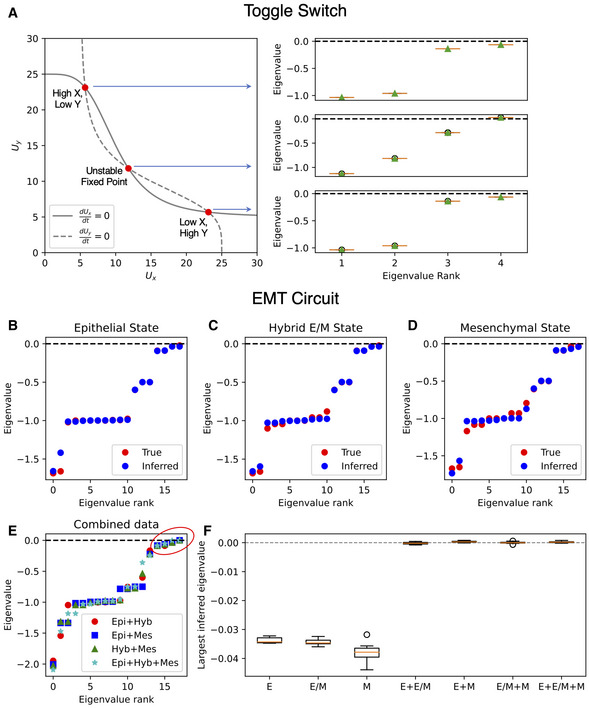
Spectral analysis of unstable fixed points A
Left: The phase space of the toggle switch synthetic circuit. Right: The eigenvalues of the Jacobian matrices in the three fixed points, ranked in ascending order, inferred by spliceJAC.B–D
The ground truth (red) and inferred (blue) eigenvalue spectrum in the three fixed points of the EMT circuit.E
The inferred eigen‐spectrums cells sampled from multiple cell states are merged. Positive eigenvalues in the red circle indicate instability.F
Comparison of largest eigenvector inferred by spliceJAC in the three stable states and in the cell mixtures. The boxplot central bands and boxes depict average, first to third quantile (Q1–Q3) range, respectively, while the whisker extension corresponds to 1.5× the interquartile range (IQR). The boxplot results are computed over 10 independent simulations.

We further consider a biologically relevant scenario and generalize a circuit that regulates epithelial–mesenchymal transition (EMT) model (Tian *et al*, 2013) by explicitly including mRNA splicing (Appendix Methods, section 3). This circuit includes two epithelial microRNAs (miR‐34 and miR‐200), two mesenchymal transcription factors (ZEB and SNAIL), an external TGF‐beta signal that induces EMT, the cellular TGF‐beta, and two output nodes for the epithelial (E‐cadherin) and mesenchymal (N‐cadherin) phenotypes, respectively (Fig 2E). Increasing TGF‐beta levels induce a transition from an epithelial state, passing through hybrid E/M state, and finally to a mesenchymal state (Fig 2F). Given our interest in capturing the context‐specific dynamics of multistable gene regulatory networks, we select an inducer level resulting in coexistence of the three states (Fig 2F, red dashed line). From a biological standpoint, this corresponds to a scenario where epithelial, hybrid E/M, and mesenchymal states are accessible. Following the simulation scheme employed for the toggle switch and 3‐gene circuit, we robustly recover the interaction matrices associated with the epithelial, hybrid E/M, and mesenchymal states (Fig 2G and H; Appendix Fig S1). To test spliceJAC's ability to discern between stable and unstable cell states, we simulated short trajectories around the unstable fixed point of the toggle switch synthetic circuit, where spliceJAC correctly predicts a positive eigenvalue that is indicative of instability (Fig EV1A). Furthermore, to “simulate” an erroneous mixing of states that could happen in real datasets, we ran spliceJAC on mixtures of cells belonging to multiple cell states of the EMT circuit. In this case, spliceJAC always predicted the largest eigenvalue to be either positive or extremely close to zero (Fig EV1B–F). Overall, these simulations demonstrate spliceJAC's ability to distinguish between stable and unstable states from simulation of synthetic circuits. Moreover, the inference was relatively robust even when genes were removed, one at a time, from the spliceJAC count matrix input, and the inference was run on the remaining subset of genes (Appendix Fig S2). This approach confirms the inference robustness *in silico* when missing genes might play important regulatory roles.

Finally, we compare spliceJAC to other existing tools for GRN inference (Huynh‐Thu *et al*, 2010; Kim, 2015; Chan *et al*, 2017; Matsumoto *et al*, 2017; Specht & Li, 2017; Gao *et al*, 2018; Sanchez‐Castillo *et al*, 2018; Woodhouse *et al*, 2018; Moerman *et al*, 2019; Aubin‐Frankowski & Vert, 2020; Deshpande *et al*, 2022) using the Beeline pipeline (Pratapa *et al*, 2020). The methods' performance for interaction recovery in the three states was quantified based on element‐wise absolute difference between the ground truth and estimated Jacobians (Fig 2I) and fraction of incorrect signs in the estimated Jacobian (Fig 2J). spliceJAC consistently performed better in both metrics and in all three states. To test diverse biological scenarios, we further used the BoolODE simulation tool to generate synthetic data for three cases: a cycling circuit exhibiting a limit cycle, a bifurcating converging circuit with two stable states, and a trifurcating circuit with three stable states (Appendix Fig S3) previously studied in the Beeline GRN inference benchmarking package (Methods and Protocols: Simulation of *in silico* circuits). To quantify the goodness of state‐specific GRN inference, we used the Beeline package to evaluate the area under the precision recall curve (AUPRC, Methods and Protocols: Benchmarking and comparison with existing GRN inference methods), showing that spliceJAC consistently achieved better scores for all the simulated synthetic circuits (Fig 2K; Appendix Table S1). While the AUPRC calculation does not penalize incorrect sign detection, the inference of the limit cycle highlights spliceJAC's ability to correctly predict not only the circuit topology but also the correct signs for activation or inhibition (Appendix Fig S4). While the existing inference methods were designed to predict a “global” gene regulatory network without considering potential major differences among different cell states, spliceJAC is specifically tailored to capture cell state‐specific interactions. Overall, the benchmarking analysis reveals the robust performance of spliceJAC to infer gene regulations.

### Identification of cell state‐specific regulatory interactions in the pancreas epithelium

**Figure 3:**
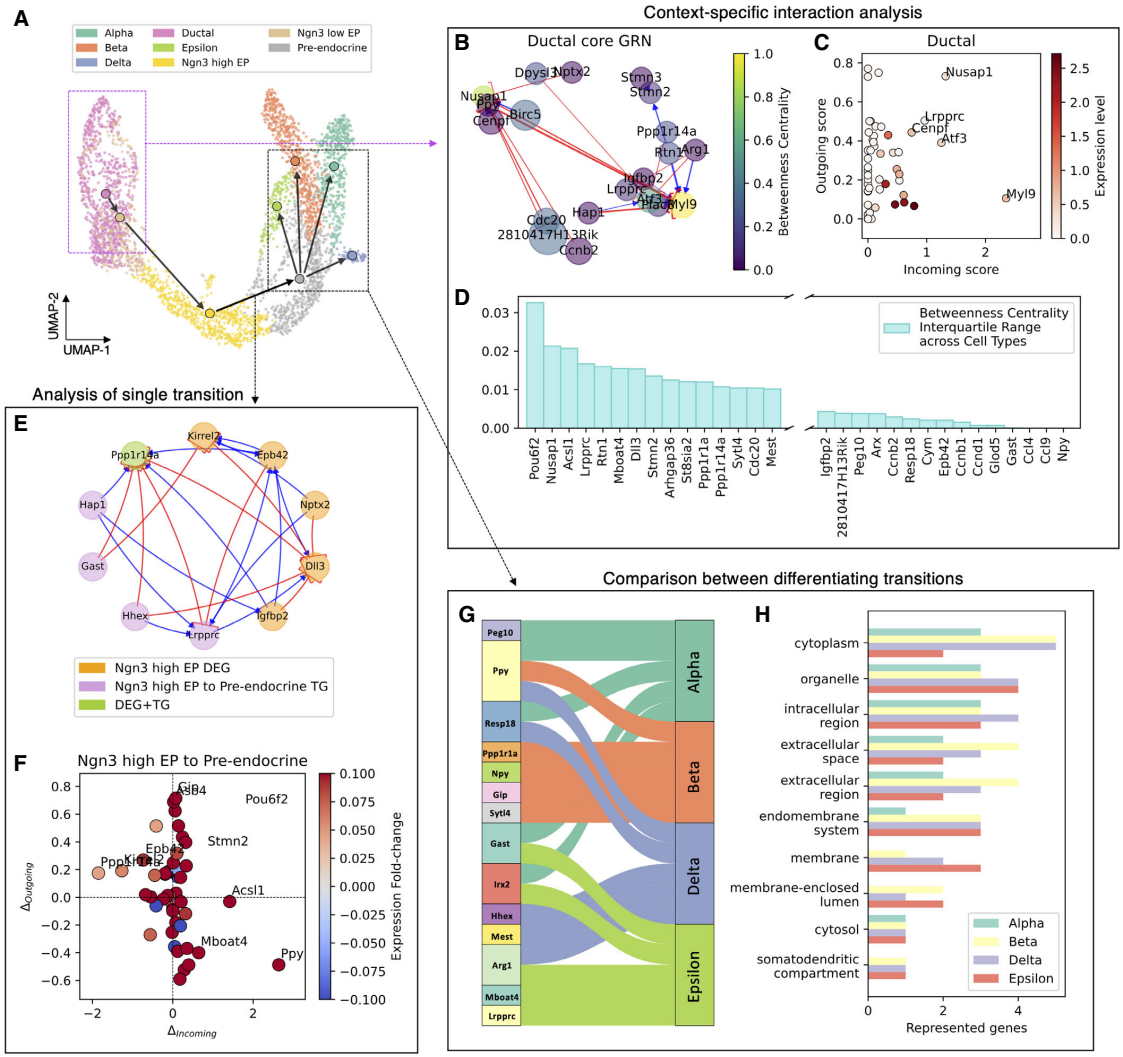
Identification of cell state‐specific regulatory interactions in the pancreas epithelium UMAP embedding of the pancreas epithelium dataset from Bastidas‐Ponce *et al* (2019). Arrows indicate cell transitions identified with PAGA.A core gene regulatory network governing gene expression in Ductal state. Node size depicts gene expression level within the Ductal cluster while the color scale depicts betweenness centrality of the node.Scatterplot of the genes based on incoming interaction strength (x‐axis) and outgoing interaction strength (y‐axis).State‐to‐state variability of gene betweenness centrality across cell states quantified by the interquartile range. Leftmost genes have highest state‐to‐state variability, whereas rightmost genes have lowest state‐to‐state variability.A core GRN including the top Differentially Expressed Genes (DEG) of the Ngn3 high EP cell state and the top Transition Genes (TG) for the transition toward the Pre‐endocrine state.Change in incoming and outgoing signaling scores during the transitioning from Ngn3 high EP to pre‐endocrine.Flowchart highlighting the shared and unique Transition Genes (TG) for differentiation from Pre‐endocrine to Alpha, Beta, Delta, and Epsilon, respectively.Gene Ontology analysis for the top five identified transitioning genes leading to the Alpha, Beta, Delta, and Epsilon cluster, respectively.

In order to gauge spliceJAC on an experimental dataset, we consider scRNA‐seq data from the mouse pancreas epithelium development (Bastidas‐Ponce *et al*, 2019). Previous analysis of transition trajectories with PAGA and RNA velocity modeling uncovered a transition from Ductal precursor cells and the final differentiation between four terminal cell states: Alpha, Beta, Delta, and Epsilon (La Manno *et al*, 2018; Wolf *et al*, 2019; Fig 3A). Delta represents the rarest population, with only 70 identified cells, thus putting an upper limit to the number of genes considered in the spliceJAC inference (Methods and protocols: Analysis of pancreas epithelium data).

First, we investigate the context‐specific gene–gene interactions, resulting in a series of core gene regulatory networks (GRN) that encode context‐specific interactions within each cell state (Fig 3B; Appendix Fig S5A). The gene regulatory networks associated to each cell state can be visualized with varying levels of detail based on quantile weight selection feature (Appendix Fig S5B). The reconstructed interaction network enables the analysis of the gene signaling roles, which can be quantified with several different metrics, including betweenness centrality of the gene node in the GRN, overall incoming and outgoing signaling through the gene node, and the “total signaling” defined as the sum of incoming and outgoing signaling scores (Methods and Protocols: Quantification of signaling roles and signaling change upon transition). The main signaling hubs can be identified as the genes with higher incoming and outgoing signaling scores (Fig 3C; Appendix Fig S6). Moreover, comparing gene signaling roles across cell states provides information about genes with highly specialized or conserved functions. For each gene, the variability in signaling role can be tested in spliceJAC with three separate metrics, including standard deviation (SD), range, and interquartile range (Methods and Protocols, section 3). For example, inspecting the interquartile range of betweenness centrality across cell states highlights a group of genes with high state‐to‐state variability, such as Pouf62, Acsl1, Nusap1, Dll3, and Lrpprc (Fig 3D). We further test the robustness of the gene–gene interaction inference method by exploring its consistency, response to subsampling, and prediction with different regression methods and parameters (Appendix Fig S7).

**Figure EV2:**
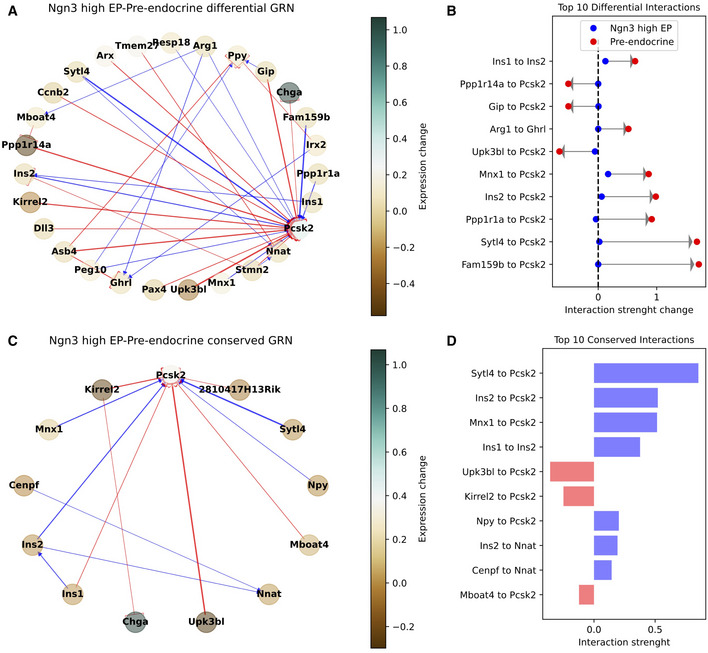
The differential and conserved interactions in the Ngn3 high EP/Pre‐endocrine cell state transition A
The differential GRN between the Ngn3 high EP and Pre‐endocrine cell states. Node colormap indicates the gene expression fold‐change between the cell states.B
The top 10 differential interactions between the Ngn3 high EP and Pre‐endocrine cell states. Arrows depict the interaction strength change.C, D
The conserved GRN (C) and top 10 conserved interactions (D) highlight the interactions that maintained similar strength from the Ngn3 high EP to the Pre‐endocrine cell state.

**Figure EV3:**
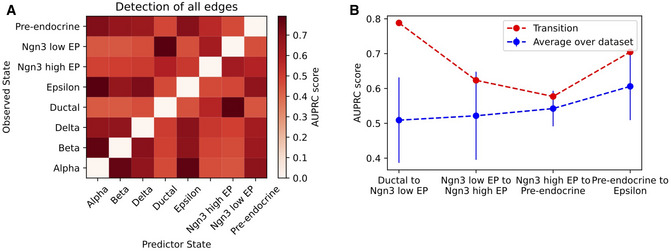
The GRN similarity along a developmental trajectory The area under the precision‐recall curve (AUPRC) when predicting the GRN of the observed GRN (*y*‐axis) using the GRN of the predictor state (*x*‐axis).The goodness of GRN prediction along a developmental trajectory. For each point, the red point shows the AUPRC between the GRNs of the starting and final state. For comparison, the blue dots and error bars show the average and standard deviation (SD) of AUPRC obtained when comparing the GRN of the starting state to the GRN of any other state in the dataset (*n* = 6 other states in the dataset excluding the final state).

Moreover, the state transition analysis offers information about the most unstable genes that can destabilize cell states and drive transitions. Applying this analysis to the pancreas epithelium lineage predicts key transition genes (TGs) associated with each transition (Appendix Fig S8A). Interestingly, comparing these “instability scores” with standard differentially expressed gene (DEG) scores highlights that DEGs of the starting cell state tend to have above average instability scores, thus confirming their important role in the gene regulation of the specific cell state. Other genes with high instability score were identified, however, that do not stand out based on their DEG score, thus potentially offering additional information on the specific transition dynamics that is not captured by existing analysis pipelines (Appendix Fig S8A). We further compared spliceJAC's instability scores with the cluster‐specific top‐likelihood genes identified by scVelo's dynamical modeling, again confirming that many genes are uniquely predicted by spliceJAC (Appendix Fig S8B). The key regulatory interactions between DEGs and TGs can be summarized in a core GRN (Fig 3E; Appendix Fig S9A). Moreover, comparing the GRNs of starting and final cell states suggests key genes with substantial changes in their signaling role during a specific transition (Fig 3F; Appendix Fig S9B). The implications of cell state transition on gene regulation can be captured by differential and conserved GRNs that rank the top gene–gene interactions with largest or smallest change between the starting and final states (Fig EV2). It may be expected that adjacent cell states share more similarities in GRN structure compared with the states that are not directly connected by a transition. Comparing the GRNs along a developmental trajectory from the Ductal to the Alpha state shows that the GRNs of adjacent cell states along the transition retain more similarity compared with GRNs of nonadjacent cell states, as quantified by the area under the precision‐recall curve (AUPRC). In other words, the GRNs of the “starting states” are typically good predictors for the GRNs of the “final states” (Fig EV3). The consistency of GRN inference and transition gene analysis was further confirmed when randomly removing subsets of genes from the dataset, which mimics missing genes that can either be undetected in the dataset or not selected for the spliceJAC analysis (Appendix Fig S10).

Finally, the transition analysis can be generalized to lineages where an initial cell state differentiates into multiple different final states, as seen in the differentiation of pre‐endocrine cells into specific endocrine subtypes, including Alpha, Beta, Delta, and Epsilon (see black dashed box in Fig 3A). The transition analysis of all these switches can be merged to identify important TGs that are exclusive to a single differentiation branch or shared between multiple differentiation branches (Fig 3G). Moreover, specific genes that are selectively active in only one of the final endocrine subtypes can be visualized based on a boxplot (Appendix Fig S9C and D). To gain biological insight into the role of these genes, we further perform gene ontology (GO, Methods and protocols: Gene Ontology analysis) analysis of the top five transition genes leading to the four final cell states (Fig 3H). This analysis highlights the involvement in distinct biological processes of genes that drive transitions toward different terminal states. For example, the genes associated to the Beta state are well‐represented in extracellular space regulation, whereas Delta genes are more represented in the intracellular region process.

Together, spliceJAC suggests that the significant topological changes in gene regulations during pancreas epithelium development result in the coordinated alternations of transcriptome profiles, which shapes the directions of transition paths on cell state bifurcation landscape.

### Signaling changes and driver genes during partial and complete EMT in A549 lung carcinoma cells

**Figure 4:**
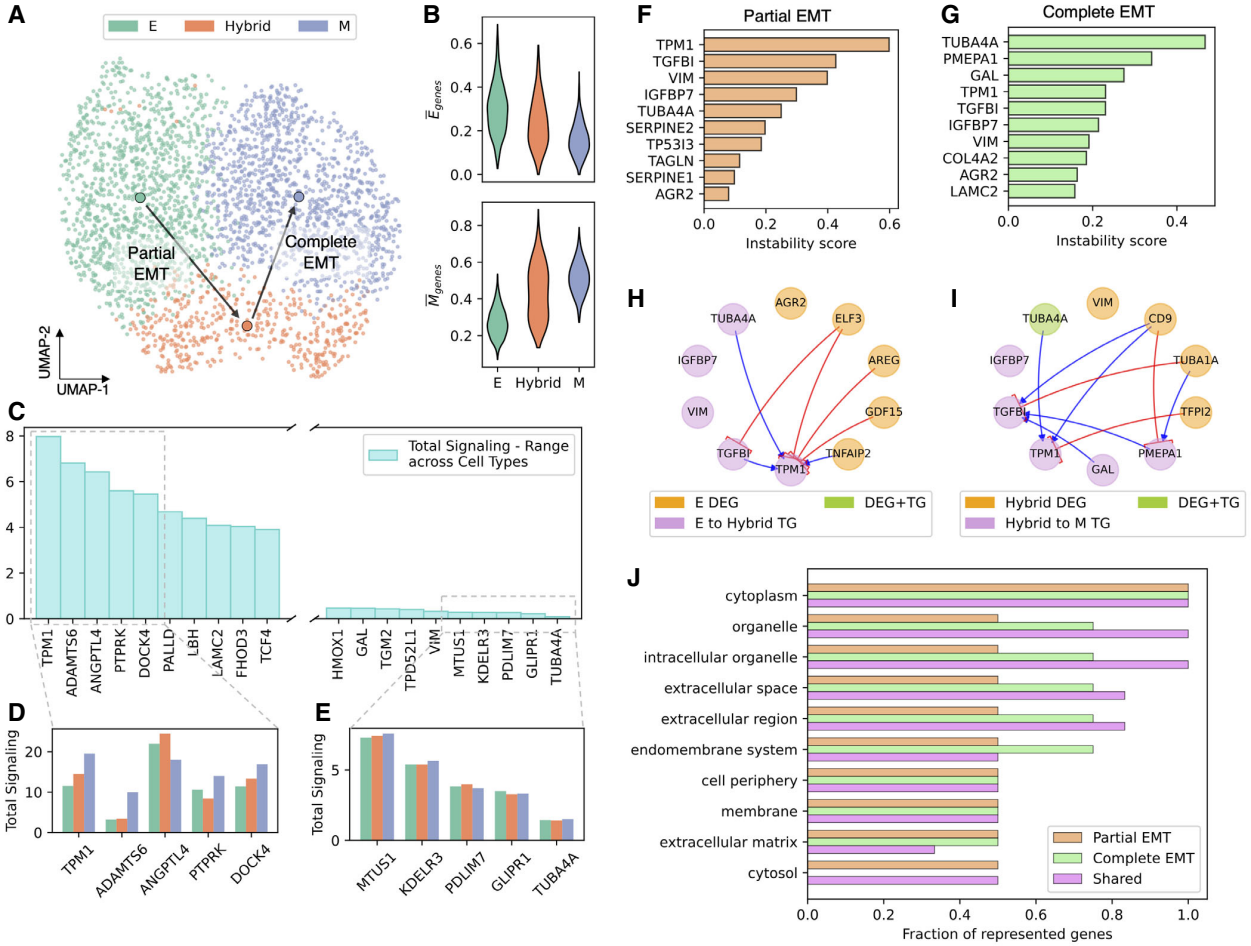
Analysis of partial and complete EMT in A549 cells UMAP embedding of the A549 cells under TGFB induction(Cook & Vanderhyden, 2020). Arrows indicate cell transitions for partial and complete EMT.Average expression of epithelial and mesenchymal genes in the three identified cell states.Variability of total signaling across cell states quantified by the total signaling range across the three identified cell states. Leftmost genes have high state‐to‐state variability in their regulatory behavior, whereas rightmost genes have low state‐to‐state variability.Detail for the top five variable genes identified in panel (C). The bar plot showcases the total signaling of the genes in each of the three cell states.Same as (D) for the top five least variable genes.Top 10 transitioning genes in the “partial EMT” transition from epithelial to hybrid E/M states.Same as (F) for the “complete EMT” transition from hybrid E/M to mesenchymal states.A core GRN indicating the inferred interactions between top differentially expressed genes (DEG) of the epithelial state and the top transition genes of partial EM Transition.Same as (H) for the complete EMT transition.Gene Ontology analysis for the top 10 identified transitioning genes for partial and complete EMT. The analysis compares transitioning genes that are exclusive to partial EMT, exclusive to complete EMT, and shared between the two transitions.

Next, we consider a time course of A549 lung carcinoma epithelial cells (*n* = 3,003 cells) *in vitro* (Cook & Vanderhyden, 2020) where epithelial–mesenchymal transition (EMT) was induced by a constant dosage of TGFβ, and scRNA‐seq was performed at successive time points (t = 0 days, 8 h, 1 day, 3 days). Since we are specifically interested in the signaling changes associated with EMT‐related genes, we restrict our analysis to a list of well‐known epithelial and mesenchymal genes implicated in TGFβ‐driven EMT (Foroutan *et al*, 2017), resulting in *n* = 25 epithelial genes and *n* = 102 mesenchymal genes. Trajectory inference on scRNA‐seq time course datasets has previously shown that TGFβ induces transitions to a mesenchymal state that pass through one or more intermediate, or hybrid epithelial/mesenchymal (E/M) cell states (Bocci *et al*, 2021; Sha *et al*, 2021). Therefore, we choose clustering parameters resulting in three clusters corresponding to different cell states (Fig 4A and Methods and Protocols: Analysis of A549 cell line under TGF‐beta induction). Two clusters exhibit clear epithelial and mesenchymal traits, having, respectively, high/low expression of epithelial genes and low/high expression of mesenchymal genes, while a third cluster expresses both groups of genes at intermediate levels, and it is thus identified as a hybrid E/M state (Fig 4B).

First, gene–gene interaction inference on the selected epithelial and mesenchymal gene sets uncovers the specific regulatory interactions of EMT‐related genes. While it is generally difficult to benchmark gene regulatory network predictions, mesenchymal genes tend to suppress the expression of epithelial genes while often activating other mesenchymal genes. Similarly, epithelial genes tend to activate other epithelial genes while repressing mesenchymal genes (Dongre & Weinberg, 2019). Consistently, we find a promising trend in all cell states where regulation from E to M genes and *vice versa* is mostly negative, whereas regulations between epithelial genes or between mesenchymal genes are mostly positive (Appendix Fig S11). Moreover, the global analysis of gene signaling role across clusters identifies genes with selectively high signaling activity in different clusters (Fig 4C–E). For example, many mesenchymal genes such as TMP1, ADAMST6, PTPRK, and DOCK4 exhibit highest signaling scores in the mesenchymal state. Other genes, such as ANGPTL4, exhibit maximal signaling activity in the hybrid E/M state and might perhaps be associated with a partial, rather than a complete EMT (Fig 4D).

Finally, we compare transitions from epithelial to hybrid (or “partial EMT”) and from hybrid to mesenchymal (or “complete EMT”). The analysis reveals key genes driving partial and complete EMT (Fig 4F and G) and their connection with the differentially expressed genes in the epithelial and hybrid E/M cell states (Fig 4H and I). Interestingly, a fraction of transition genes is shared between partial and complete EMT, including TMP1, TGFBI, VIM, IGFBP7, and AGR2. To gain biological insight into the role of these genes, we performed Gene Ontology analysis (Wu *et al*, 2021) on the top 10 transition genes identified for the partial and complete transitions (Methods and protocols: Gene Ontology analysis). Interestingly, the shared TGs play an important role in the organelle and extracellular region processes, whereas the complete EMT genes are more highly represented in endomembrane systems (Fig 4J). Overall, most biological processes in the GO analysis exhibit similar representation for partial and complete EMT transition genes, perhaps unexpectedly when considering that these are two steps of the same trans‐differentiation process.

To sum up, our analysis highlights the specific role of transition‐driver genes that regulate the partial and complete EMT process and regulate distinct biological processes.

## Discussion

We have presented spliceJAC, a method to analyze the attractor basin structures of gene expression and RNA splicing dynamics, which allows to predict critical transition driving genes and cell state‐specific interactions between genes. Compared with existing frameworks for mRNA splicing and gene–gene reconstruction methods, spliceJAC incorporates multiple kinetic regimes characterized by state‐specific kinetic parameters, thus enabling to tackle complex scenarios where multiple, stable cell states can coexist. Moreover, it considers a multivariate mRNA splicing framework where the production of nascent mRNA is regulated by other species. This key feature enables to obtain information about the stability of cell states, and prediction of “unstable” genes that induce state transitions. The identification of transition driver genes is rooted in the theory of attractor linear stability, thus providing a potentially more interpretable alternative to existing approaches to single‐cell transcriptomics. Together, spliceJAC has three important functions that allow novel biological insights: (i) distinguishing transition driver genes leading to different final states that stem from a common initial state, such as the differentiation of pre‐endocrine cells into either Alpha, Beta, Delta, and Epsilon cells in during pancreas endocrinogenesis; (ii) characterizing gene regulation in intermediate cell states that are not transitory but rather act as stable attractors, such as the hybrid epithelial/mesenchymal state during EMT; and (iii) quantifying the differences in gene signaling roles between distinct cell states.

Reconstructing interactions between genes from single‐cell transcriptomic data, and scRNA‐seq data specifically, is an important problem that has been tackled with various techniques (Pratapa *et al*, 2020; Nguyen *et al*, 2021). Learning causal relations between genes can unlock novel biological insights and even predict the effect of molecular perturbations such as mutations or therapeutic interventions (Heydari *et al*, 2022). Several available GRN inference methods, such as SCODE (Matsumoto *et al*, 2017), SCNS (Woodhouse *et al*, 2018), and GRISLI (Aubin‐Frankowski & Vert, 2020), rely on pseudotime ordering. When considering only cells from a unique cell state, however, it is reasonable to assume that pseudotime variations between cells are small. Therefore, while these methods are useful in biological situations where intermediate cell states do not satisfy a steady state assumption, spliceJAC describes scenarios where multiple stable attractors exists, as seen for instance in reversible EMT (such as during wound healing) and in many diseases such as cancer (Brabletz *et al*, 2018; Mercedes *et al*, 2021). Furthermore, a second class of methods such as GINIE3 (Huynh‐Thu *et al*, 2010) and GRNboost2 (Moerman *et al*, 2019) assumes steady state, similarly to spliceJAC; the lack of dynamical information provided by the unspliced/spliced mRNA classification, however, potentially leads to incorrect sign estimation and often hampers the ability to predict causal relations between genes (Appendix Methods, section 4). Finally, the recent RNA‐ODE employs the existing RNA velocity framework to calculate a gene force field, from which a gene regulatory network and transition paths are inferred(Liu *et al*, 2022).

Moreover, the majority of existing GRN inference methods focuses on “global” gene–gene interaction networks. Recently, other interesting methods started to focus on the cell state specificity of gene regulation. The cell type‐specific gene networks in the developing fetal brain and in autistic patient samples has been developed, relying on a local independent test, which is carried out on a cell type‐basis (Wang *et al*, 2021); GRNs of distinct cell types have been constructed by integrating prior knowledge and gene activity (Gibbs *et al*, 2022); a fine‐grained method has been developed to infer cell‐specific networks (CSN) and predict important genes that are neglected by traditional differential gene expression analysis (Dai *et al*, 2019). One common trait of these methods is to adjust the global GRN inference strategies, such as correlation, mutual information, and regression, and apply them to specific cells or subpopulations of cells (Akers & Murali, 2021). In the present work, the terms “cell state” and “cell type” are largely considered as synonymous. While the term “cell state” has a clear relation with well‐defined mathematical concepts (such as attractors and stability), the term “cell type” is more loosely defined, and types are usually associated with expression of well‐known markers. spliceJAC users can adjust their analysis by modifying the cell annotations input to spliceJAC, for instance by merging or excluding certain cell states based on the prior knowledge on the specific biological system.

Existing methods identify important genes that play relevant roles during cell transitions based on gene expression—such as differentially expressed genes (DEG; Soneson & Robinson, 2018; Wang *et al*, 2019)—and trajectory inference—such as genes that are highly variable during transitions (Sha *et al*, 2020). Conversely, spliceJAC predicts transition driver genes based on the instability inferred from the cell state's Jacobian matrix. Our analysis of the pancreas endothelium and EMT in A549 cells showed a partial overlap between spliceJAC and standard DEG analysis, but also predicted novel transition driver genes that could not be identified with existing techniques, thus offering predictions that can be tested experimentally.

Finally, we acknowledge several aspects to further improve the model and analysis of spliceJAC. First, incorporating additional layers of datasets such as chromatin accessibility or proteomics (Gorin *et al*, 2020) could significantly enhance the accuracy of gene‐interaction terms. Furthermore, prior knowledge of gene interactions may help to reduce the false positives of regulation recovery (Dong *et al*, 2022). In the future, these additional measurements can be incorporated with spliceJAC to generate more data‐driven and robust predictions. Moreover, spliceJAC models linear interactions between genes, an assumption motivated by the linear stability theory. Both technical and biological noise, however, might result in significant deviations between cells of same state. The stochastic and dynamical modeling of gene regulation and splicing, compared with deterministic and steady‐state approaches in spliceJAC, could in the future improve the goodness of fitting toward low‐expressed genes and strengthen the inference of gene auto‐regulations (preprint: Wang & He, 2022). Finally, like other models of gene regulation based on scRNA‐seq data, spliceJAC assumes that mRNA counts provide a consistent approximation for protein copy numbers, which might not necessarily be true due to post‐translations regulations.

Overall, spliceJAC represents a step forward in the biophysical modeling of scRNA‐seq data and a power tool that unlocks novel biological insights about the context‐specificity and stability of multistable biological systems.

## Materials and Methods

### Reagents and tools

**Table:** 

Reagent/Resource	Reference or Source	Identifier or Catalog Number
**Software**		
Numpy v1.23.2	Harris *et al* (2020)	N/A
Matplotlib v3.5.3	Hunter (2007)	N/A
Pandas v1.4.4	McKinney (2010)	N/A
Networkx v2.8.6	Hagberg *et al* (2008)	N/A
Scipy v1.9.9	Virtanen *et al* (2020)	N/A
Plotly v5.10.0	Plotly Technologies Inc (2015)	N/A
Scanpy v1.9.1	Wolf *et al* (2018)	N/A
scVelo v0.2.4	Bergen *et al* (2020)	N/A
BoolODE	Pratapa *et al* (2020)	N/A
Beeline	Pratapa *et al* (2020)	N/A

### Methods and protocols

#### Inference of cell state‐specific gene–gene interactions from multivariate mRNA splicing analysis

Based on the local mRNA splicing model of equation (5a) and (5b), and assuming steady state around a given fixed point, equation (5a) and (5b) can be set to zero, and the unspliced RNA counts for gene *i* is (assume β=1 for simplicity):

(6) $${U_{i}}^{c}=A_{i0}+\sum_{j\neq i}A_{\mathrm{ij}}{S_{j}}^{c}$$

For each gene (i), the basal constant ($A_{i0}$) and set of coefficients $A_{\mathrm{ij}}$ are calculated using linear regression:

(7) $${A_{i0}}^{*},\left\{{A_{\mathrm{ij}}}^{*}\right\}=\min_{A_{i0},\left\{A_{\mathrm{ij}}\right\}}\sum_{c}{\left(A_{i0}+\sum_{j\neq i}A_{\mathrm{ij}}{S_{j}}^{c}-\beta_{i}{U_{i}}^{c}\right)}^{2}+\lambda F\left(A_{i0},\left\{A_{\mathrm{ij}}\right\}\right)$$

where all cells (denoted by sub/superscript c) within the specific cell state are considered. The input unspliced and spliced mRNA counts can be recovered from raw data using publicly available scRNA‐seq processing pipelines including Velocyto and Kallisto (La Manno *et al*, 2018; Melsted *et al*, 2021). Compared with equation (5a), the summations in equations (6) and (7) do not consider the self‐regulatory term $A_{\mathrm{ii}}$ to avoid possible collinearity caused by the linear relationship between $U_{i}$ and $S_{i}$ in equation (5b).

Repeating the regression procedure for each gene yields a gene–gene interaction matrix A whose entry $A_{\mathrm{ij}}$ represents the causal, non‐symmetric regulation from gene j to gene i. For robust parameter estimation, the spliceJAC package offers linear, ridge and lasso regressions as potential options for the shrinkage term $F\left(A_{i0},\left\{A_{\mathrm{ij}}\right\}\right)$. In the cases of ridge and lasso, the shrinkage coefficient λ can be set by the user (default λ=1). To further enhance the robustness of the inferred interaction coefficients, spliceJAC infers the interaction matrix (A) multiple times (default n=10) using bootstrapping strategy, each time randomly selecting only a fraction of cells in the cell state (default ρ=0.9), and then averaging the results for ensemble estimation. The value of all inference parameters can be adjusted by the user.

It is worth noting that the set of considered genes can be selected automatically based on standard metrics (such as highly expressed genes) or provided by the user as a list. Regardless of the selection method, the number of cells within each cell state provides an upper bound to the size of the selected gene list. In a dataset with N selected genes, each gene is characterized by N parameters that represent the regulation from the other N–1 genes (with the addition of a basal production rate). Therefore, at least N independent observations (i.e., cells) are required within each cell state to guarantee a unique solution to the regression problem. spliceJAC includes a built‐in checkpoint to determine to maximum number of genes that can be considered in the inference.

#### Quantification of signaling roles and signaling change upon transition

For any given cell state (k), the “Incoming” and “Outgoing” signaling scores are defined as the weighted sums of incoming and outgoing edges in the inferred gene regulatory network (GRN). In other words, the incoming and outgoing scores of gene i in cell state k are defined as the sums of the absolute‐valued i‐th row and i‐th column of the gene–gene interaction matrix of cell state k ($A^{k}$):

(8a) $${I_{i}}^{k}=\sum_{i=1}^{N}¦A_{\mathrm{ij}}^{k}¦$$

(8b) $${O_{i}}^{k}=\sum_{j=1}^{N}¦A_{\mathrm{ji}}^{k}¦$$

where the absolute value prevents large positive and negative matrix term from canceling each other. The “total signaling” score is defined as the sum of Incoming and Outgoing score. From the interaction matrix $A^{k}\;$, an interaction graph is constructed using the NetworkX python library. To quantify the topological importance of genes in GRN, the betweenness centrality of each gene is calculated from the graph using the built‐in NetworkX centrality method (Hagberg *et al*, 2008).

#### Signaling variability of individual genes across cell states

The signaling variability of a given gene quantifies whether the gene retains a similar signaling role or instead assumes different roles across cell states. The signaling role in each cell state can be defined based on incoming signaling, outgoing signaling, total signaling, or betweenness centrality (all defined in the previous section). Therefore, a vector $g_{i}$ with K elements, where K is the number of cell states in the dataset, is constructed for each gene i. The comparison across clusters is based on three different methods: standard deviation, range, and interquartile range. Standard deviation simply computes the standard deviation of $g_{i}$; the range is defined as the difference between largest and smallest elements of $g_{i}$; and interquartile range is defined as the length of the middle 50% interval of space in the distribution of $g_{i}$ elements. In the spliceJAC workflow, both the measure of signaling and the comparison method can be selected by the user.

#### Top differential/conserved interactions and GRN comparison between cell states

Given the gene–gene interactions matrices of two cell states, the top differential/conserved interactions are identified based on maximal/minimal absolute change in edge weight. Moreover, the similarity between GRNs of two cell states connected by a transition is quantified using AUROC/AUPRC scores, where the GRN of the final cell state is considered as “ground truth” and the GRN of the starting cell state is considered as the observable. With this definition, the AUROC/AUPRC score quantifies how well the GRN of the starting cell state predicts the GRN of the final cell state. This definition is generalized to pairs of states that are not connected by a transition to generate a similarity matrix $S\in {\mathbb{R}}^{K\times K}$ where K is the number of cell states in the dataset.

#### Identification of transition driver genes from Jacobian eigen‐space analysis

Given a transition from an initial cell state toward a final cell state, the transition driving genes are identified based on the initial state's Jacobian spectral properties and path connecting the two cell states. In general, for the mRNA splicing model of equation (5a) and (5b) with N selected genes, the Jacobian matrix is a 2N × 2N matrix to account for both unspliced and spliced mRNA species. The Jacobian matrix of the starting state $J\in {\mathbb{R}}^{2N\times 2N}$ is reconstructed from the gene–gene interaction matrix A and inference of spliced mRNA degradation rates (Appendix Methods, section 2). From linear stability theory of dynamical systems, the attractor stability of equations (3a) and (3b) or equivalently (5a) and (5b), and associated stable or unstable manifold (i.e., possible directions to make the transition) can be analyzed through the eigen‐space of Jacobian J. We propose to quantify the importance of genes during cell state transition based on such theory.

First, the spectral analysis of the Jacobian matrix associated with the starting cell state provides information about the possible unstable directions, or unstable manifold, of the state. The set of most unstable directions is defined by the eigenvectors $v_{m}\in {\mathbb{R}}^{2N}$ of the Jacobian matrix associates with the top largest eigenvalues (default n=10 largest eigenvalues).

(9) $$Jv_{m}=\lambda_{m}v_{m},\lambda_{1}\geq \lambda_{2}\geq \ldots \geq \lambda_{n}\ldots \geq \lambda_{2N}$$

It should be noted that, depending on the geometry of the attractor, all or some of the selected eigenvalues can be positive (i.e., the cell state is a saddle point) or they can all be negative (i.e., the cell state is a truly stable fixed point). This set of unstable directions is then projected onto the transition path connecting the starting and final cell states, as schematically illustrated in Fig 1D. The initial and final cell states (identified from pseudotime, dimensionality or prior knowledge) are assumed to be connected by a linear path in the gene space, so that the path displacement vector can be defined as $\Delta x=\left(U_{k},S_{k}\right)-\left(U_{w},S_{w}\right)$, where w and k denote the starting and final cell states, respectively. The unstable manifold projection onto the transition path ensures that, even though unstable eigenvectors might point in different directions in the gene space, the directionality of cell state transition is preserved. The spliceJAC package features a built‐in function to analyze transitions identified with the PAGA package(Wolf *et al*, 2019).

We then decompose the actual transition Δx from data into the n top unstable (aka transition) directions of state w:

(10) $$\Delta x\approx \sum_{m=1}^{n}\left\langle \Delta x,v_{m}\right\rangle v_{m}=\sum_{m=1}^{n}k_{m}v_{m}$$

where $\left\langle ∙\right\rangle$ denotes inner product.

Finally, the instability score $IS_{j}$ of each gene *j* is defined as the sum of squared loadings score of the gene on the eigenvector projection (including both unspliced and spliced components) on the transition path, that is,

(11) $$IS_{j}=\sum_{m=1}^{n}k_{m}^{2}\left(v_{m,j}^{2}+v_{m,j+N}^{2}\right)$$

where $v_{m,j}$ denotes the j‐th element of $v_{m}$. Intuitively, a gene with large instability score indicates its large loadings in the unstable directions that are significant in the actual transitions.

#### Simulation of *in silico* circuits

The bistable toggle switch, monostable 3‐gene circuit, and tristable EMT circuit (Tian *et al*, 2013) were simulated with stochastic differential equations and using custom‐built code (Appendix Methods, section 3). In each case, we simulate small perturbations around each stable fixed point. First, the system is initialized exactly on the fixed point; afterward, a small, gaussian perturbation is added. Finally, the relaxation trajectories are merged and provided as input to the spliceJAC inference function.

The cycle, bifurcating converging, and trifurcating circuits were simulated with the BoolODE package downloaded from https://github.com/Murali‐group/BoolODE. All simulation parameters were fixed based on their original values. Input files and simulation results for BoolODE simulations are deposited at https://github.com/cliffzhou92/jacobian‐inference‐benchmarking.

#### Benchmarking and comparison with existing GRN inference methods

We used the Beeline pipeline (Pratapa *et al*, 2020) to benchmark the performance of existing GRN inference algorithms on the EMT, cycle, bifurcating and trifurcating synthetic dataset. In each algorithm, the GRNs were inferred for all stable states and/or limit cycle separately, for a total of nine cell states. For each state, spliced gene counts were taken as the input gene expression matrices, since existing algorithms do not take RNA splicing process into account. For the cycle, bifurcating and trifurcating circuits simulated with BoolODE, both mRNA and protein counts were provided as inputs for spliceJAC inference, corresponding to the unspliced and spliced counts. For algorithms that also require pseudotime ordering as the input, we calculated the cell's individual diffusion pseudotime time (Haghverdi *et al*, 2016) in each state using the scanpy (Wolf *et al*, 2018) package, with the root selected as cell expressing minimal value of E‐cad gene, and hyper‐parameters selected as *n*_neighbors = 4 and *n*_pcs = 20. All other algorithm parameters were set as default as in Beeline pipeline.

For the EMT circuit, the Jacobian matrices computed analytically were used as reference ground truth. For the cycle, bifurcating, and trifurcating circuits, a ground truth Jacobian could not be generated analytically. Therefore, the circuit network topologies were used as ground truth, were positive and negative edges were assigned weights of 1 and −1, respectively. The goodness of GRN inference was quantified using Beeline's built‐in area under the operator‐receiver curve (AUROC) and area under the precision‐recall characteristic (AUPRC). All Beeline parameters for AUROC/AUPRC calculation were fixed to their default values. All Beeline input and output files are deposited at https://github.com/cliffzhou92/jacobian‐inference‐benchmarking. Moreover, we quantified the inference on the EMT circuit based on (i) absolute difference and (ii) fraction of correct signs between ground truth and inferred GRN matrices. To compute the absolute difference, both ground truth ($J_{\text{true}}$) and inferred ($J_{\inf}$) GRN matrix elements are normalized between −1 and 1; the absolute difference is then defined as the element‐wise sum of $¦J_{\text{true}}-J_{inf}¦$.

#### Analysis of pancreas epithelium data

The dataset for pancreas epithelium development was originally generated by Bastidas‐Ponce *et al* (2019) and was directly obtained through the scVelo library with the datasets.pancreas() function (Bergen *et al*, 2020). The size of the Delta cell population (*n* = 70 cells) defines the upper limit for the number of selected genes for spliceJAC inference. Moreover, since spliceJAC employs a bootstrapping method where the gene–gene interaction inference is repeated multiple times (*n*sim = 10 by default) using only a fraction *p* of cells (*p* = 0.9 by default), the inference is limited to a theoretical maximum of *pn* = 63 genes. Therefore, the 50 top highly expressed genes were selected. spliceJAC inference and downstream analysis were performed on the selected dataset.

#### Analysis of A549 cell line under TGF‐beta induction

The time course of A549 cells under TGF‐beta induction performed by Cook & Vanderhyden (2020) were downloaded from the GSE repository under the accession number GSE147405. The original dataset already included counts matrices for unspliced and spliced mRNAs. To focus the analysis on EMT‐related genes, we selected genes from epithelial and mesenchymal gene signatures previously identified in the context of TGFβ‐driven EMT (Foroutan *et al*, 2017), resulting in a selected dataset with 127 total genes (*n* = 25 epithelial genes and *n* = 102 mesenchymal genes). spliceJAC inference and downstream analysis were performed on the selected dataset. Cell clusters were identified with the Scanpy Leiden clustering algorithm; a resolution parameter of r = 0.3 was chosen resulting in three clusters. The average expression of epithelial and mesenchymal genes presented in Fig 4B in each cluster was computed as the expression average of all epithelial/mesenchymal genes in all the cluster cells.

#### Gene Ontology analysis

Gene Ontology analysis was performed using the ClusterProfiler R library (Wu *et al*, 2021). For the GO analysis of the pancreas epithelium transitioning genes, GO analysis was executed on the top five transitioning genes leading to transitions from the Pre‐endocrine state toward the Alpha, Beta, Delta, and Epsilon states, respectively. Results were aggregated and plotted together (Fig 3H) for direct comparison between transitions. For the GO analysis of the A549 cell line, the top 10 predicted transitioning genes were selected for the partial EMT and complete EMT transitions. The genes were divided into three categories: (i) gene specific to partial EMT transition, (ii) genes specific to complete EMT transition, and (iii) genes shared by the two transitions. GO analysis was executed for the three gene sets separately, and results were normalized based on the number of genes in each gene set before comparison.

#### Software availability

spliceJAC is available as a python package at https://github.com/federicobocci/spliceJAC.

## Author contributions

**Federico Bocci:** Conceptualization; data curation; formal analysis; investigation; methodology; writing – original draft; writing – review and editing. **Peijie Zhou:** Conceptualization; data curation; formal analysis; supervision; investigation; methodology; writing – review and editing. **Qing Nie:** Resources; supervision; funding acquisition; project administration; writing – review and editing.

## Disclosure and competing interests statement

The authors declare that they have no conflict of interest.

## Supporting information



Appendix S1

Expanded View Figures PDF

PDF+

## Data Availability

This study did not generate new data. The pancreas endocrinogenesis dataset is available in Gene Expression Omnibus under accession number GSE132188 (http://www.ncbi.nlm.nih.gov/geo/query/acc.cgi?acc=GSE132188). The A549 time series dataset is available in Gene Expression Omnibus under the accession GSE147405 (http://www.ncbi.nlm.nih.gov/geo/query/acc.cgi?acc=GSE147405). The spliceJAC package and source code are available at: https://github.com/federicobocci/spliceJAC. The benchmarking scripts and results are available at: https://github.com/cliffzhou92/jacobian‐inference‐benchmarking.
