# Supplementary material for: spliceJAC: transition genes and state‐specific gene regulation from single‐cell transcriptome data
Source: Mol Syst Biol. 2022 Nov 2;18(11):e11176. doi: 10.15252/msb.202211176 (PMC9627675; doi:10.15252/msb.202211176)
Supplement: Supplementary file 1 — Appendix S1 [file MSB-18-e11176-s004.pdf]

Appendix

spliceJAC: Transition genes and state specific gene regulation from single-cell transcriptome data

Federico Bocci[1,3], Peijie Zhou[1,*], Qing Nie[1,2,3*]

[1]Department of Mathematics, University of California, Irvine, CA 92697, USA
[2]Department of Developmental and Cell Biology, University of California, Irvine, CA 92697, USA
[3]NSF-Simons Center for Multiscale Cell Fate Research, University of California, Irvine, CA 92697, USA

*Authors for correspondence:
Peijie Zhou (peijiez1@uci.edu)
Qing Nie (qnie@uci.edu)

Table of contents

Appendix Methods……………………………………………………………………….2

1. Model derivation for pairwise interactions……………………………………………2
2. Calculation and inference of the Jacobian matrix……………………………………..2
3. Generation of in silico data………………………………………………………………3

   3.1 Bistable toggle switch…………………………………………………………..3

   3.2 Three gene circuit……………………………………………………………….4

   3.3 EMT core circuit………………………………………………………………..4

4. Relations with existing GRN inference methods based on spliced mRNA…………...6

Supplementary references…………………………………………………………………7

Appendix Figures (S1-S11)……………………………………………………………….8

Appendix Tables (S1)……………………………………………………………………...19

Appendix Methods

1. Model derivation for pairwise gene interactions

A multivariate model of mRNA splicing is written based on eqs. (3) from the main text as:

$$\frac{dU_i}{dt} = k_i f(S_1, S_2, \dots, S_N) - \beta U_i \qquad (1a)$$

$$\frac{dS_i}{dt} = \beta U_i - \gamma_i S_i \quad (1b)$$

Where U_i and S_i are the copy numbers for unspliced and spliced mRNA of species i, and $f(S_1, S_2, \dots, S_N)$ is the gene-gene interaction function. Often, systems biology modeling of gene regulatory networks assumes pairwise interactions between genes. In this case, eqs. (1) assume the form:

$$\frac{dU_i}{dt} = k_i \prod_{j=1}^{N} H^i(S_j) - \beta U_i \qquad (2a)$$

$$\frac{dS_i}{dt} = \beta U_i - \gamma_i S_i \quad (2b)$$

In eq. (2a), the production rate is modeled as a product of individual functions describing the regulatory action of all other species (S_j) on U_i. Typically, each species is directly regulated only by a few other species, and thus the product is simplified to contain only a few terms. It is assumed here that each species regulates its targets in an independent fashion, which might not be true in specific circumstances where different transcription factors cooperate to activate or inhibit their target gene. From eqs. (2), each individual gene regulation function can be linearly expanded around stable fixed points:

$$H^i(S_j) \approx H^i(S_j{}^{ss}) + C_{ij}(S_j - S_j{}^{ss}) + O\left(\left(S_j - S_j{}^{ss}\right)^2\right) \quad (3)$$

Substituting the linear expansion of eq. (3) back into eq. (2a) and discarding quadratic and higher-order terms yields a linear expansion with the same function form of the one derived in main text, eq. (5a). The relation between the gene-gene interaction coefficients in main text, eq. (5a), and the expansion coefficients of eq. (3) is:

$$A_{ij} = C_{ij} \prod_{k \neq i} H^i(S_k{}^{ss}) \quad (4)$$

In other words, the expansion coefficient C_{ij} is multiplied by the steady state value of all pairwise interaction functions.

2. Calculation and inference of the Jacobian matrix

Around each fixed point, the multivariate mRNA splicing model can be linearly approximated and expressed in the compact form:

$$\frac{dU_i}{dt} = \alpha_{i0} + \sum_{j=1}^{N} A_{ij} S_j - \beta U_i = F_U{}^i(\boldsymbol{U}, \boldsymbol{S}) \quad (1a)$$

$$\frac{dS_i}{dt} = \beta U_i - \gamma_i S_i = F_S{}^i(\boldsymbol{U}, \boldsymbol{S}) \quad (1b)$$

Where $\boldsymbol{U}$ and $\boldsymbol{S}$ are vectors of unspliced and spliced mRNA count, respectively. The Jacobian matrix associated with eqs. (1) can be divided into four major block matrices with the following forms:

$$\frac{\partial F_U{}^i(\boldsymbol{U},\boldsymbol{S})}{\partial U_j} = -\beta\delta_{ij} \quad (2a)$$
$$\frac{\partial F_U{}^i(\boldsymbol{U},\boldsymbol{S})}{\partial S_j} = A_{ij} \quad (2b)$$
$$\frac{\partial F_S{}^i(\boldsymbol{U},\boldsymbol{S})}{\partial U_j} = \beta\delta_{ij} \quad (2c)$$
$$\frac{\partial F_S{}^i(\boldsymbol{U},\boldsymbol{S})}{\partial S_j} = -\gamma_i\delta_{ij} \quad (2d)$$

Where $\delta_{ij} = 1$ if $i = j$ and 0 otherwise. Assuming that the splicing rate coefficient β can be rescaled to unit value, eqs. (2a) and (2c) yield unitary matrices. Moreover, eq. (2b) yields the gene-gene interaction matrix that is inferred as described in Methods, section 1. Finally, eq. (2d) yields a diagonal matrix of spliced degradation rates. Degradation rates are inferred one gene at a time by solving the linear regression problem:

$$\gamma_i{}^* = \min_{\gamma_i} \sum_c (U_i{}^c - \gamma_i S_i{}^c)^2 \quad (3)$$

While the elements of the gene-gene interaction matrix (A_{ij}) are computed locally (i.e., one cell state at a time), the spliced mRNA degradation rate constants are computed globally (i.e., using all cells in the dataset), because degradation is a linear term, and the degradation rate constants should not be functions of the specific cell state.
The resulting Jacobian matrix is used to study the cell state stability and transition driver genes leading toward other cell states.

3. **Generation of in silico data**

Bistable toggle switch

The bistable toggle switch includes two transcription factors that mutually inhibit each other's expression, and is typically modeled with two coupled ordinary differential equations that describe the temporal dynamics of concentration/copy number for proteins (X, Y):

$$\frac{dX}{dt} = k_X \frac{1}{1 + \left(\frac{Y}{Y_0}\right)^n} - \gamma X \quad (1a)$$
$$\frac{dY}{dt} = k_Y \frac{1}{1 + \left(\frac{X}{X_0}\right)^n} - \gamma Y \quad (1b)$$

Where k_X, k_Y are basal production rates than integrate both transcription and translation, X_0, Y_0 are half-concentrations, n is a Hill coefficient and γ is a degradation rate. To explicitly include mRNA splicing, we distinguish between the unspliced (U_X, U_Y) and spliced (S_X, S_Y) mRNA levels:

$$\frac{dU_X}{dt} = k_X \frac{1}{1 + \left(\frac{S_Y}{Y_0}\right)^n} - \beta U_X \quad (2a)$$

$$\frac{dU_Y}{dt} = k_Y \frac{1}{1+\left(\frac{S_X}{X_0}\right)^n} - \beta U_Y \quad (2b)$$

$$\frac{dS_X}{dt} = \beta U_X - \gamma S_X \quad (2c)$$

$$\frac{dS_Y}{dt} = \beta U_Y - \gamma S_Y \quad (2d)$$

Where β is the splicing rate constant and it is assumed to be the same for all genes. In eqs. (2), the feedback regulation between X and Y is included in the unspliced mRNA production rate. Therefore, eqs. (2) assume that the spliced mRNA levels approximate well the level of transcription factors (X, Y).

Three-gene circuit

The monostable 3-node circuit is composed by three genes (X, Y, Z), where X activates Y, Y activates Z, and Z activates X. Following the same strategy of the toggle switch, the temporal dynamics of unspliced and spliced mRNA for the three species within a cell is modeled with a system of ODEs:

$$\frac{dU_X}{dt} = k_X \frac{\left(\frac{S_Z}{Z_0}\right)^n}{1+\left(\frac{S_Z}{Z_0}\right)^n} - \beta U_X \quad (3a)$$

$$\frac{dU_Y}{dt} = k_Y \frac{\left(\frac{S_X}{X_0}\right)^n}{1+\left(\frac{S_X}{X_0}\right)^n} - \beta U_y \quad (3b)$$

$$\frac{dU_Z}{dt} = k_Z \frac{\left(\frac{S_Y}{Y_0}\right)^n}{1+\left(\frac{S_Y}{Y_0}\right)^n} - \beta U_Z \quad (3c)$$

$$\frac{dS_X}{dt} = \beta U_X - \gamma S_X \quad (3d)$$

$$\frac{dS_Y}{dt} = \beta U_Y - \gamma S_Y \quad (3e)$$

$$\frac{dS_Z}{dt} = \beta U_Z - \gamma S_Z \quad (3f)$$

EMT core circuit

To model EMT, we start from the circuit developed by Tian and collaborators (Tian *et al*, 2013). This circuit includes two epithelial microRNAs (miR-34 and miR-200), two mesenchymal transcription factors (ZEB, SNAIL), which were originally modeled both at the mRNA and protein levels, an external TGF-beta signal that induces EMT, the cellular TGF-beta, and two output nodes for the epithelial (E-cadherin) and mesenchymal (N-cadherin) phenotypes, respectively. The original circuit equations were generalized to explicitly include unspliced and spliced mRNA dynamics. Specifically, each original equation is split between an unspliced term that includes production rate and splicing, and a spliced term that includes splicing and degradation:

Intracellular TGF-beta (U_T, S_T):

$$\frac{dU_T}{dt} = k0_T + \frac{k_T}{1 + \left(\frac{S_{R_2}}{J_T}\right)^{n_{r2}}} - \beta U_T \quad (4a)$$

$$\frac{dS_T}{dt} = \beta U_T - kd_T S_T \quad (4b)$$

Snail mRNA (U_s, S_s) and protein (U_S, S_S):

$$\frac{dU_s}{dt} = k0_s + k_s \frac{\left(\frac{S_T + TGF0}{J_T}\right)^{n_t}}{1 + \left(\frac{S_T + TGF0}{J_T}\right)^{n_t}} - \beta U_s \quad (5a)$$

$$\frac{dS_s}{dt} = \beta U_s - kd_s S_s \quad (5b)$$

$$\frac{dU_S}{dt} = k0_s + k_S S_s \frac{1}{1 + \left(\frac{S_{R_3}}{J_S}\right)^{n_{r3}}} - \beta U_S \quad (6a)$$

$$\frac{dS_S}{dt} = \beta U_S - kd_S S_S \quad (6b)$$

miR-34 (U_{R3}, S_{R3}):

$$\frac{dU_{R3}}{dt} = k0_3 + \frac{k_3}{1 + \left(\frac{S_S}{J1_3}\right)^{n_S} + \left(\frac{S_Z}{J2_3}\right)^{n_Z}} - \beta U_{R3} \quad (7a)$$

$$\frac{dS_{R3}}{dt} = \beta U_{R3} - kd_3 S_{R3} \quad (7b)$$

Zeb mRNA (U_z, S_z) and protein (U_Z, S_Z):

$$\frac{dU_z}{dt} = k0_z + k_z \frac{\left(\frac{S_S}{J_Z}\right)^{n_s}}{1 + \left(\frac{S_S}{J_Z}\right)^{n_s}} - \beta U_z \quad (8a)$$

$$\frac{dS_z}{dt} = \beta U_z - kd_z S_z \quad (8b)$$

$$\frac{dU_Z}{dt} = k0_Z + k_Z S_z \frac{1}{1 + \left(\frac{S_{R_2}}{J_Z}\right)^{n_{r2}}} - \beta U_Z \quad (9a)$$

$$\frac{dS_Z}{dt} = \beta U_Z - kd_Z S_Z \quad (9b)$$

miR-200 (U_{R2}, S_{R2}):

$$\frac{dU_{R2}}{dt} = k0_2 + \frac{k_2}{1 + \left(\frac{S_S}{J1_2}\right)^{n_S} + \left(\frac{S_Z}{J2_2}\right)^{n_Z}} - \beta U_{R2} \quad (10a)$$

$$\frac{dS_{R2}}{dt} = \beta U_{R2} - kd_2 S_{R2} \quad (10b)$$

E-cadherin (U_E, S_E):

$$\frac{dU_E}{dt} = k0_E + \frac{k_{E1}}{1 + \left(\frac{S_S}{J1_E}\right)^{n_S}} + \frac{k_{E2}}{1 + \left(\frac{S_Z}{J2_E}\right)^{n_Z}} - \beta U_E \quad (11a)$$

$$\frac{dS_E}{dt} = \beta U_E - kd_E S_E \quad (11b)$$

N-cadherin (U_N, S_N):

$$\frac{dU_N}{dt} = k0_N + k_{N1}\frac{\left(\frac{S_S}{J1_N}\right)^{n_S}}{1+\left(\frac{S_S}{J1_N}\right)^{n_S}} + k_{N2}\frac{\left(\frac{S_Z}{J2_N}\right)^{n_Z}}{1+\left(\frac{S_Z}{J2_N}\right)^{n_Z}} - \beta U_N \quad (12a)$$

$$\frac{dS_N}{dt} = \beta U_N - kd_N S_N \quad (12b)$$

All model's parameters were taken from the original model. The splicing rate coefficient (β) is assumed to be rescaled to unit value.

4. Relations with existing GRN inference methods based on spliced mRNA

Traditionally, the underlying dynamics for mature gene expression can be written as

$$\frac{dS_i}{dt} = k_i F_i(\boldsymbol{S}) - \gamma_i S_i \qquad (1)$$

Where, similar to main text, eq. (1), the nonlinear term $F_i(\boldsymbol{S})$ represents regulatory effects on the gene S_i. This dynamical system is equivalent to main text, eq. (3) if (a) the time scale of unspliced mRNA dynamics in main text, eq. (3a) is where quasi-stationary assumption holds, or (b) the system is close to the stationary point such that in both cases we have:

$$k_i F_i(\boldsymbol{S^{ss}}) = \gamma_i S_i^{ss} \quad (2)$$

A large class of GRN inference methods for scRNA-seq data can be interpreted as inferring the $F_i(\boldsymbol{S})$ terms in eq. (1), either dynamically or with the steady-state assumptions.

First, dynamical methods such as SCODE (Matsumoto *et al*, 2017), SCNS (Woodhouse *et al*, 2018) and GRISLI (Aubin-Frankowski & Vert, 2020), estimate time series of derivative $\widehat{\frac{dS_i}{dt}}$ for each gene by using pseudotime-ordered gene expression. Afterwards, a regression problem between dependent variable $\widehat{\frac{dS_i}{dt}}$ and variable $\boldsymbol{S} = (S_1, S_2, \ldots, S_N)$ is conducted to determine the rand-hand side of eq. (1). Biological scenarios where the pseudo-time ordering exactly reveals the single time trajectory dynamics of eq. (1) can indeed allow the recover of gene regulatory terms and is therefore consistent with our methods under assumption (a). The sensitivity to the specific pseudotime method and stochasticity or heterogeneity in scRNA-seq data, however, will pose threats for the validity of such dynamical approaches.

Second, steady-state methods assume that mRNA expression is equilibrated ($\frac{dS_i}{dt} = 0$), and therefore try to fit the term $F_i(\boldsymbol{S})$ with regression methods from eq. (2). The direct fitting, however, is not feasible because the problem is not statistically identifiable, given that $F_i(\boldsymbol{S}) = \frac{\gamma_i}{k_i} S_i$ represents the trivial solution where $F_i(\boldsymbol{S})$ is simply a diagonal matrix. Therefore, methods such as GINIE3 (Huynh-Thu *et al*, 2010) and GRNboost2 (Moerman *et al*, 2019) modify the regression problem as

$$S_i = G_i(\boldsymbol{S_{-i}}) + \epsilon_i \quad (3)$$

where $\boldsymbol{S_{-i}} = (S_1, \ldots, S_{i-1}, S_{i+1}, \ldots, S_N)$. Namely, eq. (3) assumes that S_i can be considered as a function of all other species' copy number excluding S_i itself. To understand the relations

between eq. (3) and our proposed method, we further expand eq. (1) with first-order expansion around steady state

$$\frac{dS_i}{dt} = \alpha_{i0} + \sum_{j=1}^{N} A_{ij} S_j - \gamma_i S_i \quad (4)$$

Hence, we roughly have

$$S_i \approx \frac{\alpha_{i0}}{\gamma_i - A_{ii}} + \sum_{j \neq i}^{N} \frac{A_{ij}}{\gamma_i - A_{ii}} S_j \quad (5)$$

indicating that linearization of eq. (3) is consistent with our estimation of $\boldsymbol{A}$ in terms of relative weights. However, since the ground truth of $(\gamma_i - A_{ii})$ is generally unknown from the data, this class of steady-state methods might be limited in recovering the exact signs in dynamical gene expression model.

Supplementary References

Aubin-Frankowski P-C & Vert J-P (2020) Gene regulation inference from single-cell RNA-seq data with linear differential equations and velocity inference. *Bioinformatics* 36: 4774–4780

Huynh-Thu VA, Irrthum A, Wehenkel L & Geurts P (2010) Inferring Regulatory Networks from Expression Data Using Tree-Based Methods. *PLoS One* 5: e12776

Matsumoto H, Kiryu H, Furusawa C, Ko MSH, Ko SBH, Gouda N, Hayashi T & Nikaido I (2017) SCODE: an efficient regulatory network inference algorithm from single-cell RNA-Seq during differentiation. *Bioinformatics* 33: 2314–2321

Moerman T, Santos SA, González-Blas CB, Simm J, Moreau Y, Aerts J & Aerts S (2019) GRNBoost2 and Arboreto: efficient and scalable inference of gene regulatory networks. *Bioinformatics* 35: 2159–2161

Tian XJ, Zhang H & Xing J (2013) Coupled reversible and irreversible bistable switches underlying TGFβ-induced epithelial to mesenchymal transition. *Biophys J* 105: 1079–1089

Woodhouse S, Piterman N, Wintersteiger CM, Göttgens B & Fisher J (2018) SCNS: a graphical tool for reconstructing executable regulatory networks from single-cell genomic data. *BMC Syst Biol* 12: 59

Appendix Figures

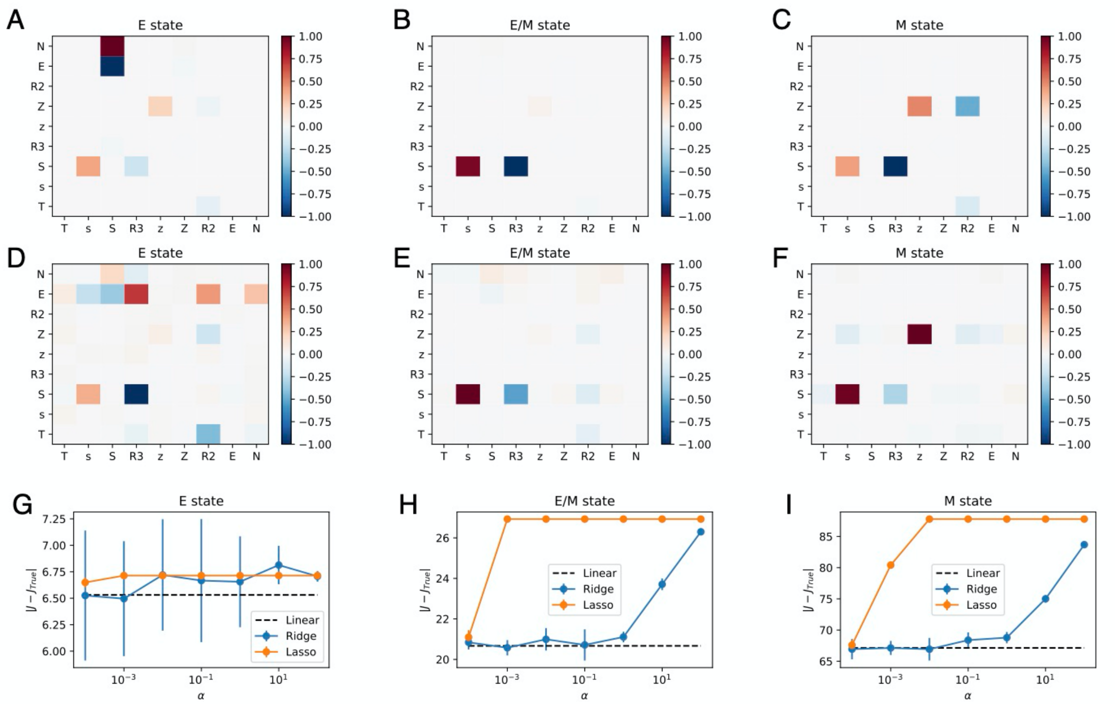

Appendix figure S1. **Inference of gene-gene interactions in the EMT circuit. (A-B-C)** Gene-gene interaction matrices for the epithelial (A), hybrid E/M (B), and mesenchymal (C) states. **(D-E-F)** Corresponding gene-gene interaction matrices inferred with spliceJAC. **(G-H-I)** Comparison between linear, ridge and lasso regression in the spliceJAC inference in the epithelial (A), hybrid E/M (B), and mesenchymal (C) states. The y-coordinate represents the deviation between ground truth and inferred matrices. The black dashed line represents the linear regression result, whereas blue and yellow data points represent results with ridge and lasso regression, respectively, as a function of the shrinkage parameter.

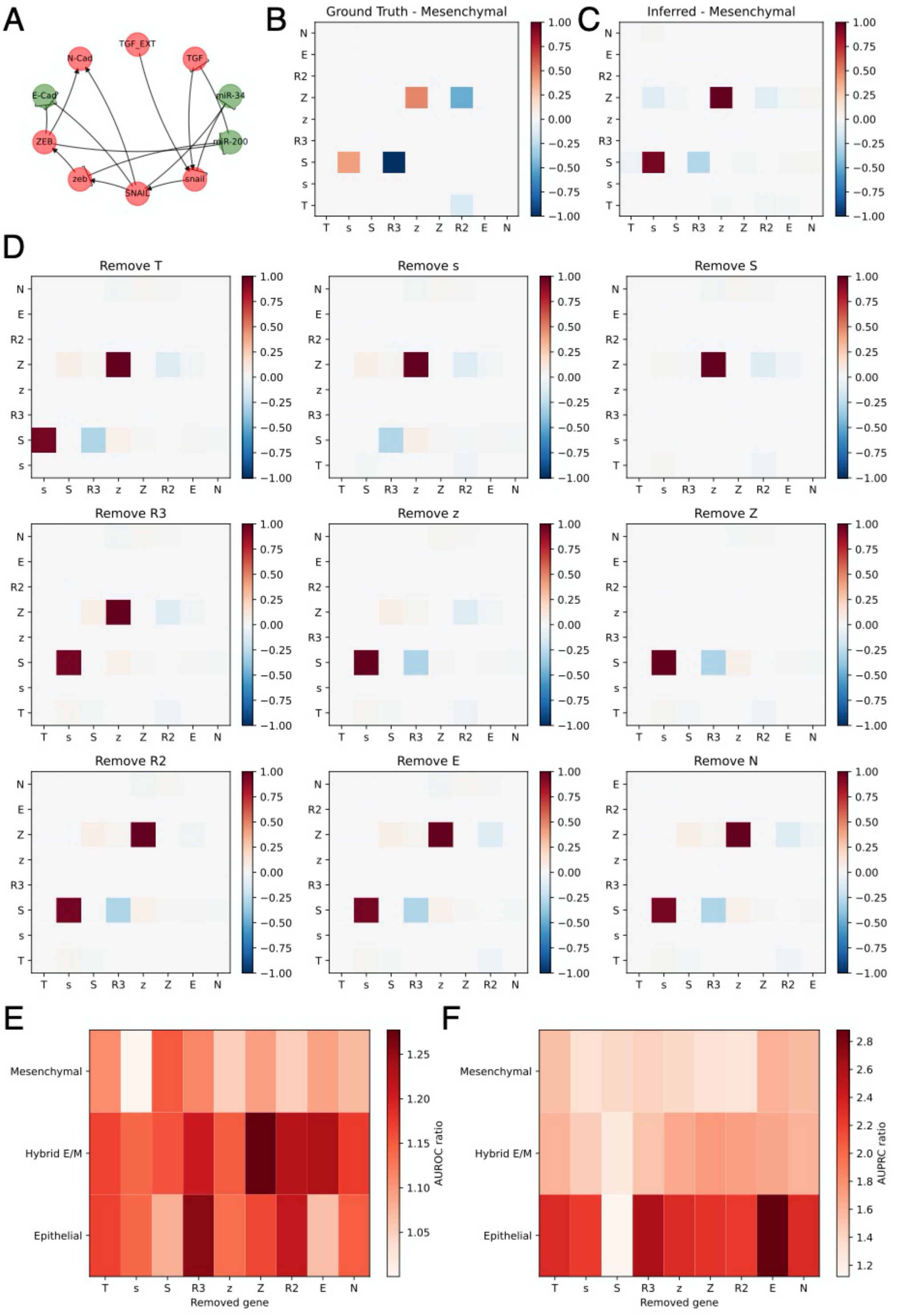

Appendix figure S2. **Inference of the EMT circuit when removing one gene at a time. (A)** The simulated EMT circuit. **(B-C)** The ground truth and inferred Jacobian matrices of the mesenchymal state (same as main Fig. 2G-F). **(D)** The inferred Jacobian matrices when removing one gene at a time from the input of the spliceJAC inference. **(E-F)** Area under the Receiver-Operator curve (AUROC) and Precision-Recall characteristic (AUPRC) to compare ground truth and inferred GRNs for the Epithelial, Hybrid E/M and Mesenchymal states when removing one gene at a time. For each combination of state/removed gene, the true value is defined as the ground truth Jacobian after removing the row and column corresponding to the removed gene; the observation is defined as the GRN inferred by

spliceJAC when omitting the expression data of the removed gene from the count matrix input. The AUC scores for each state are normalized by the AUC score obtained when considering all genes.

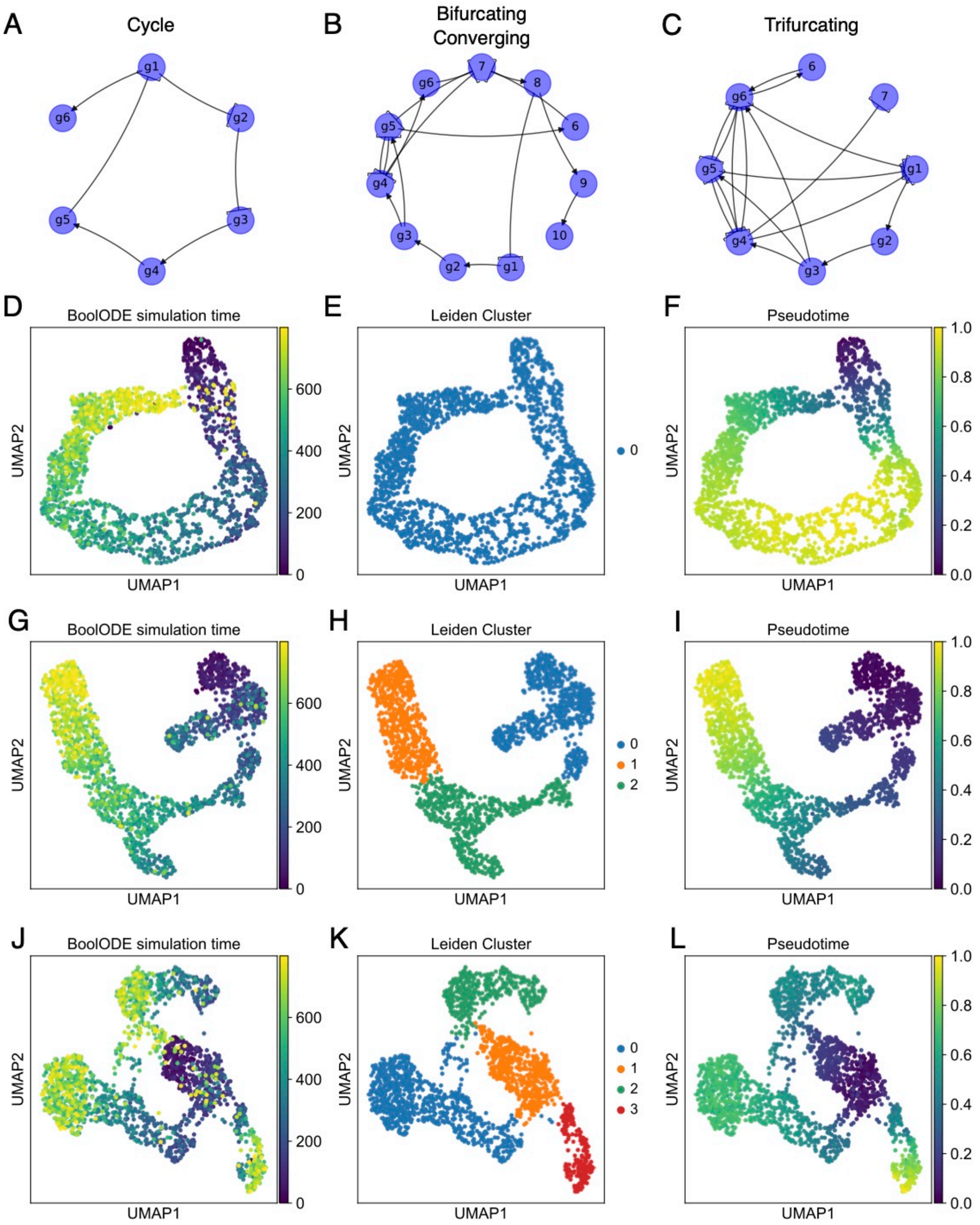

Appendix figure S3. **Simulation of synthetic circuits. (A-B-C)** The cycling, bifurcating converging, and trifurcating circuits simulated with the BoolODE pipeline. **(D)** The BoolODE simulation time for the cycling circuit. **(E)** Leiden clustering for cycling circuit consisting of a single cell state. **(F)** Pseudotime of the cycling circuit. **(G-H-I)** Same for the bifurcating converging circuit consisting of two stable cell state. **(J-K-L)** Same for the trifurcating circuit consisting of three stable cell state. The Leiden cluster labeled as "0" in panels H and K represents the initial condition state.

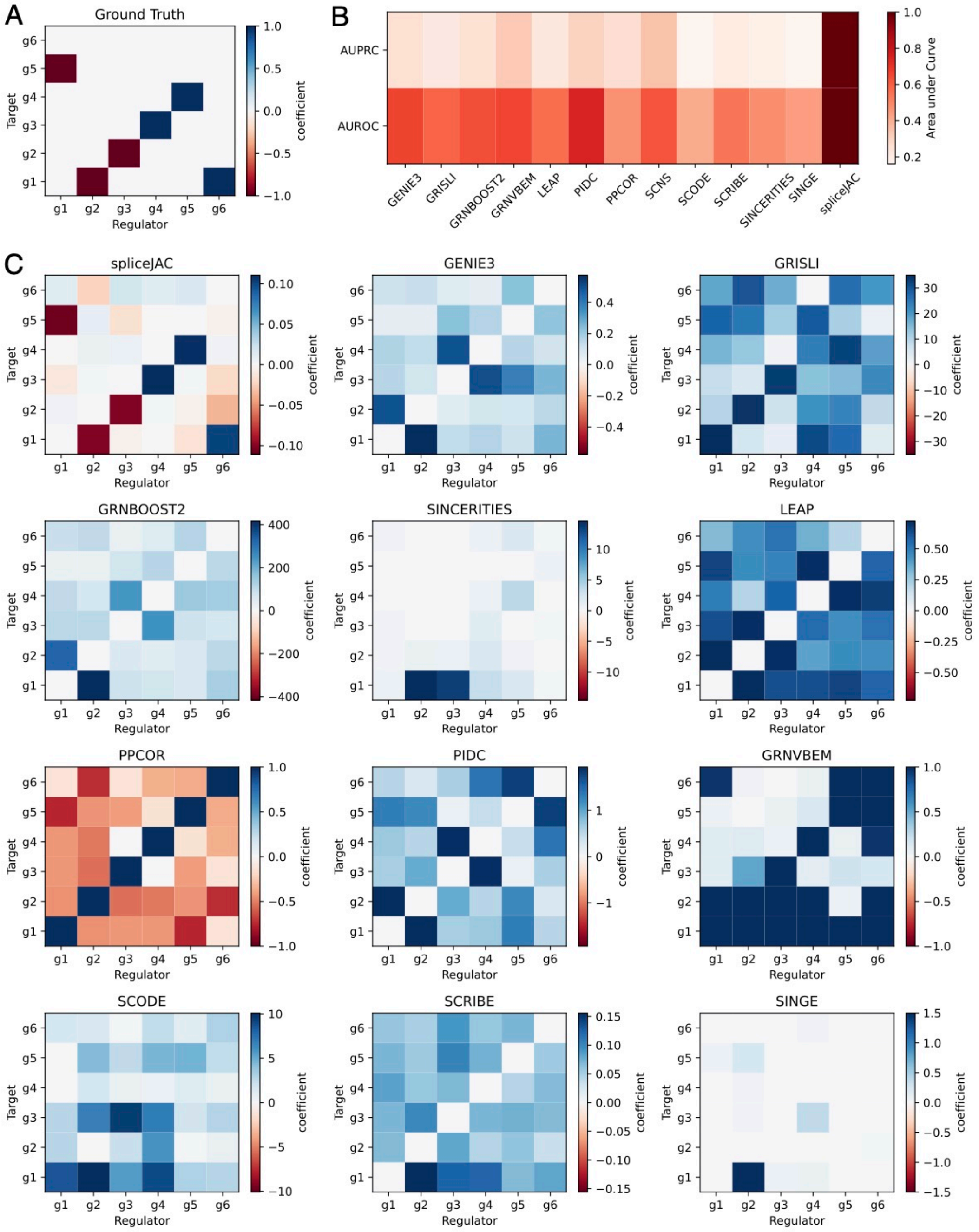

Appendix figure S4. **Inference comparison of the cycle circuit GRN. (A)** A heatmap visualization of the ground truth cycle circuit. **(B)** AUROC and AUPRC for the GRN inference methods in the Beeline pipeline and spliceJAC. **(C)** Heatmap representation of the inferred cycle GRN for all methods.

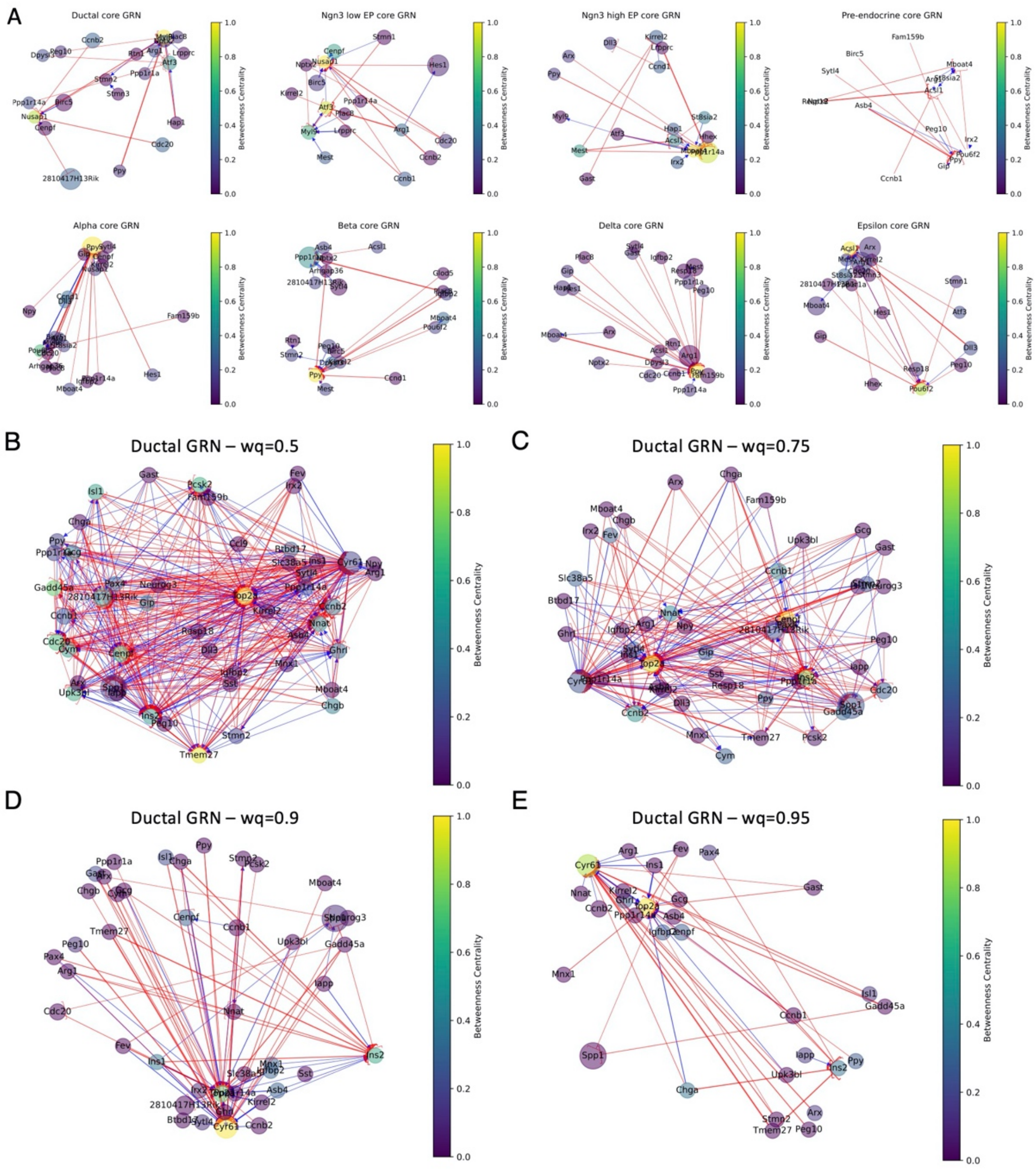

Appendix figure S5. **Cell state-specific GRNs in the pancreas endocrinogenesis dataset.** (A) Cell type-specific gene regulatory networks for the eight cell states. Node size indicates gene expression in the cell state while the color bar indicates betweenness centrality of the gene in the GRN. The plots were produced with the *visualize_network* function from the spliceJAC plotting library with a parameter weight_quantile=0.975 (i.e., only the top 2.5% interactions are plotted to highlight core GRNs). **(B-C-D-E)** The predicted gene regulatory network of the Ductal dataset for increasing weight quantile (wq) thresholds. The weight quantile parameter represents the fraction of weakest interactions that are not showed in the GRN.

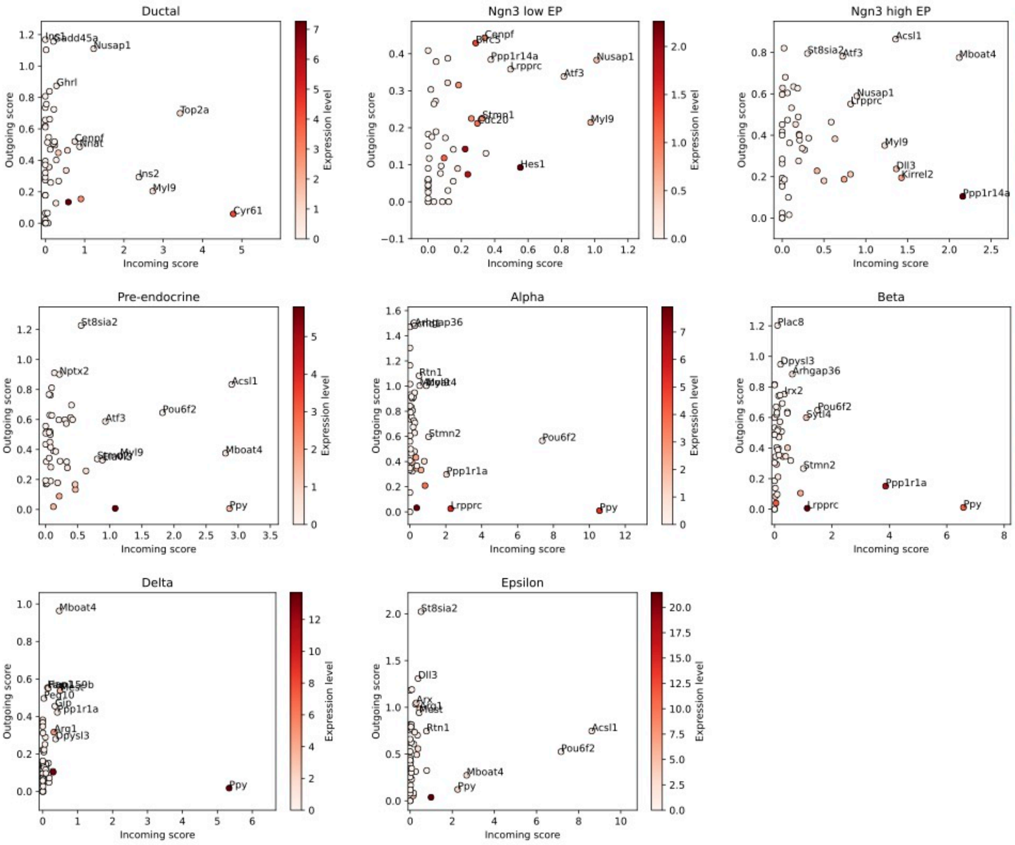

Appendix figure S6. **Cell state-specific gene signaling role.** Scatterplot of incoming and outgoing signaling scores to identify cell type-specific signaling hubs (signaling scores are defined in Methods and Protocols, section 3). The color map indicates gene expression in the cell state.

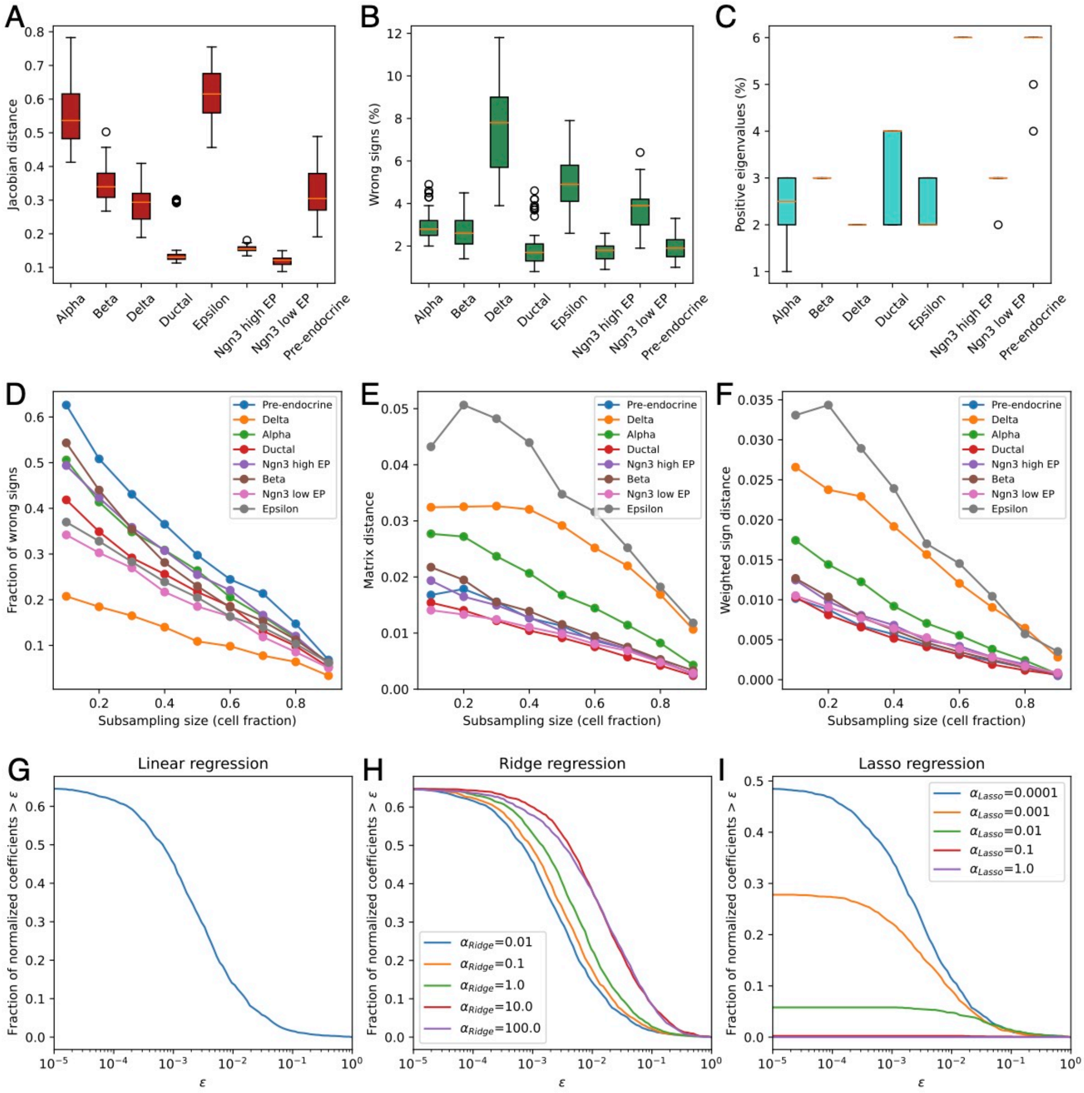

Appendix figure S7. **Robustness of the type-specific gene-gene interaction matrices.** (A-B-C) For each cell state, the Jacobian matrix is inferred n times, each time selecting only a fraction of cells (see supplementary table 1 for default parameters). The n resulting matrices are compared pairwise based on three measures: **(A)** Element-wise distance, **(B)** Percentage of inconsistent signs, and **(C)** percentage of positive eigenvalues. (D-E-F) Consistency of gene-gene interaction inference when using only a fraction of cells in the cluster. The Jacobian matrices inferred with cell subsampling are compared to the reference state-specific Jacobian matrices inferred with the standard spliceJAC pipeline (presented in Methods, section 1). The inference is compared with three metrics: **(D)** Fraction of wrong signs, **(E)** Matrix distance defined as the summation of element-wise difference, and **(F)** weighted sign distance defined as the element-wise weighted summation of differences when the incorrect interaction sign is inferred. (G-H-I) Dependence on regression method and parameters. **(G)** Fraction of gene-gene interaction matrix elements larger than a given threshold (ε) when inferring gene-gene interactions with linear regression. **(H)** Same as (G) when inferring gene-gene interactions with ridge regression. Different curves correspond to different ridge shrinkage parameters. **(I)** Same as (H) when inferring gene-gene interactions with lasso regression.

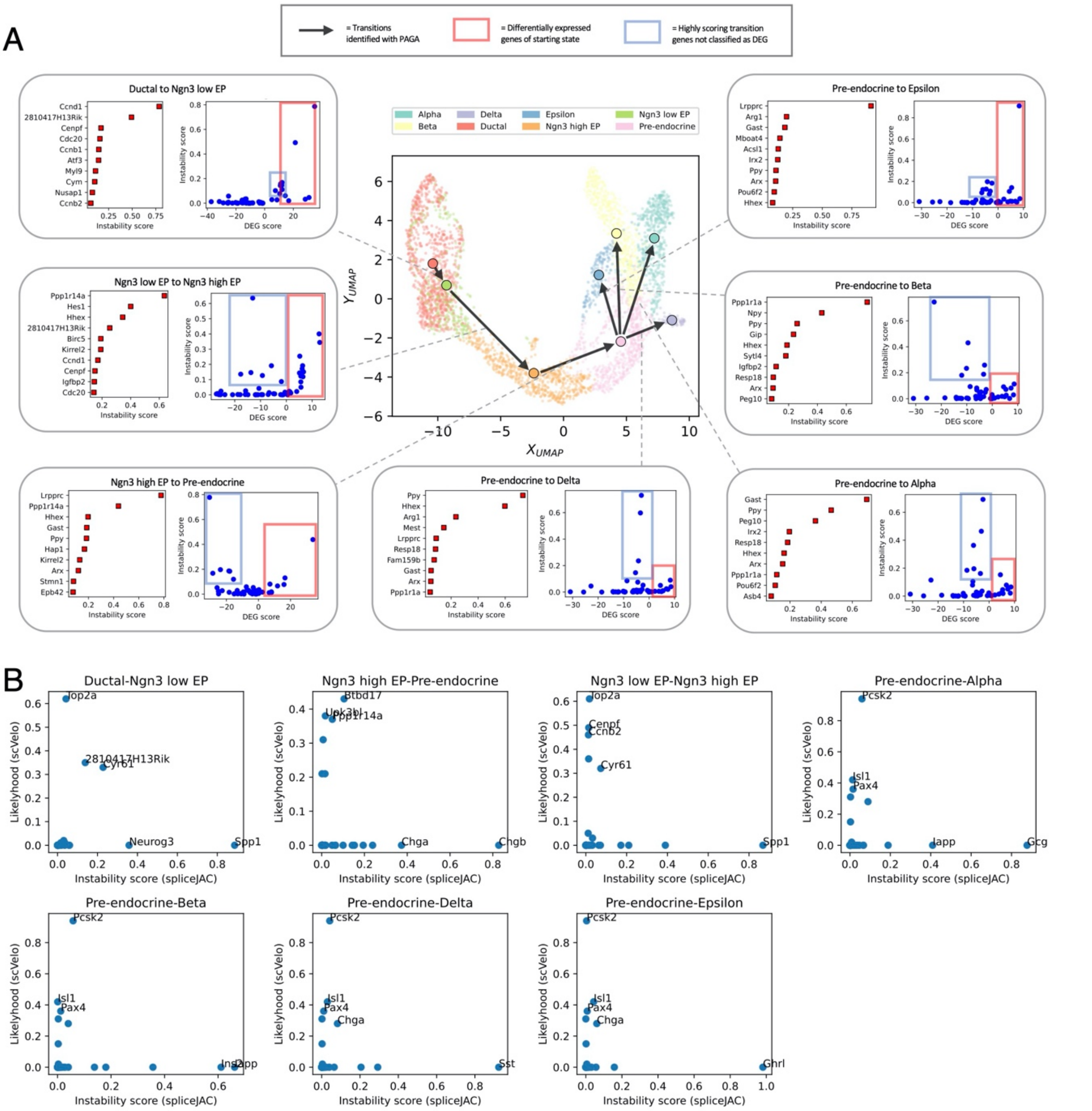

Appendix figure S8. **Analysis of instability scores in the pancreas dataset. (A)** Central panel: UMAP scatter plot of the pancreas dataset and transition trajectories identified with PAGA (same as Figure 2A). Insight panels: Top 10 genes with high instability scores for each transition (left) and gene instability score as a function of differential expression scores (DEGs) computed with the built-in Scanpy function (right). In the right insight panels, the red rectangles highlight the genes with highest DEG score (i.e., the differentially expressed genes predicted of each cell type), while the blue rectangles highlight transition genes with high instability scores that are not identified with standard DEG analysis. **(B)** Scatterplots of the transition instability score computed with spliceJAC (x-axis) and the cluster-specific top likelihood gene scores computed with scVelo.

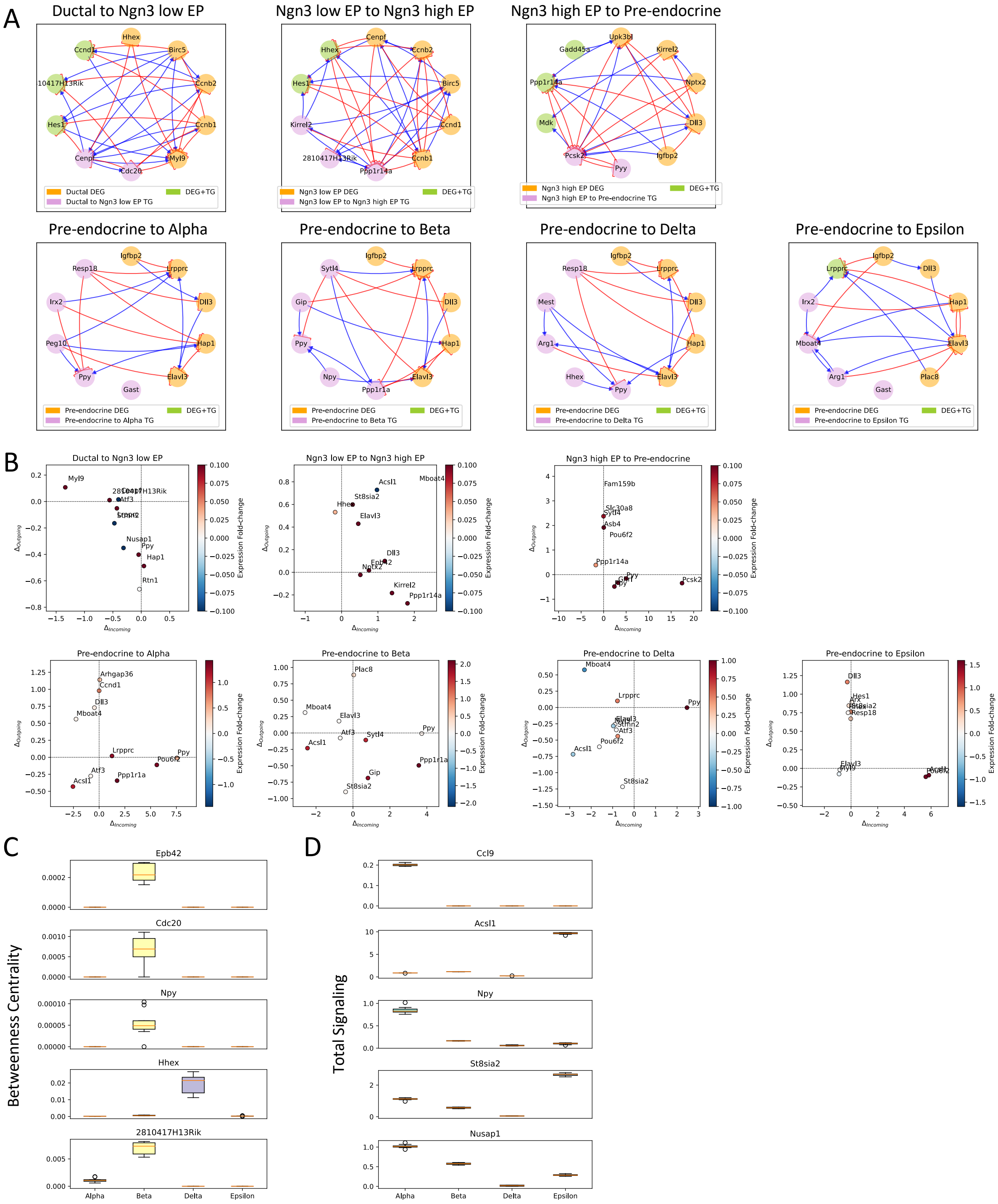

Appendix figure S9. **Transition analysis in the pancreas dataset. (A)** Core gene regulatory networks (GRNs) associated with each transition. Each GRN includes the top 5 DEG of the starting cluster (orange nodes) and the top 5 transition genes (TGs) leading to the new state (purple nodes). Green coloring highlights nodes that are both top DEG and TGs. The plots were produced with the core_GRN function from the spliceJAC plotting library. **(B)** Change in incoming and outgoing signaling scores upon transition. Labels highlight the genes with largest total score change. The plots were produced with the plot_signaling_change function from the spliceJAC plotting library. **(C-D)** Identification of genes that are selectively active only in one of the terminal cell states. The activity is quantified either with the Betweenness

centrality (C) or Total Signaling (incoming + outgoing scores, D) of the genes in the GRNs of each terminal cluster. The boxplots show measurements over n=10 separate iterations of the GRN inference method, where each time the GRNs were computed using only a randomly chosen fraction (90%) of the total cells in each cluster.

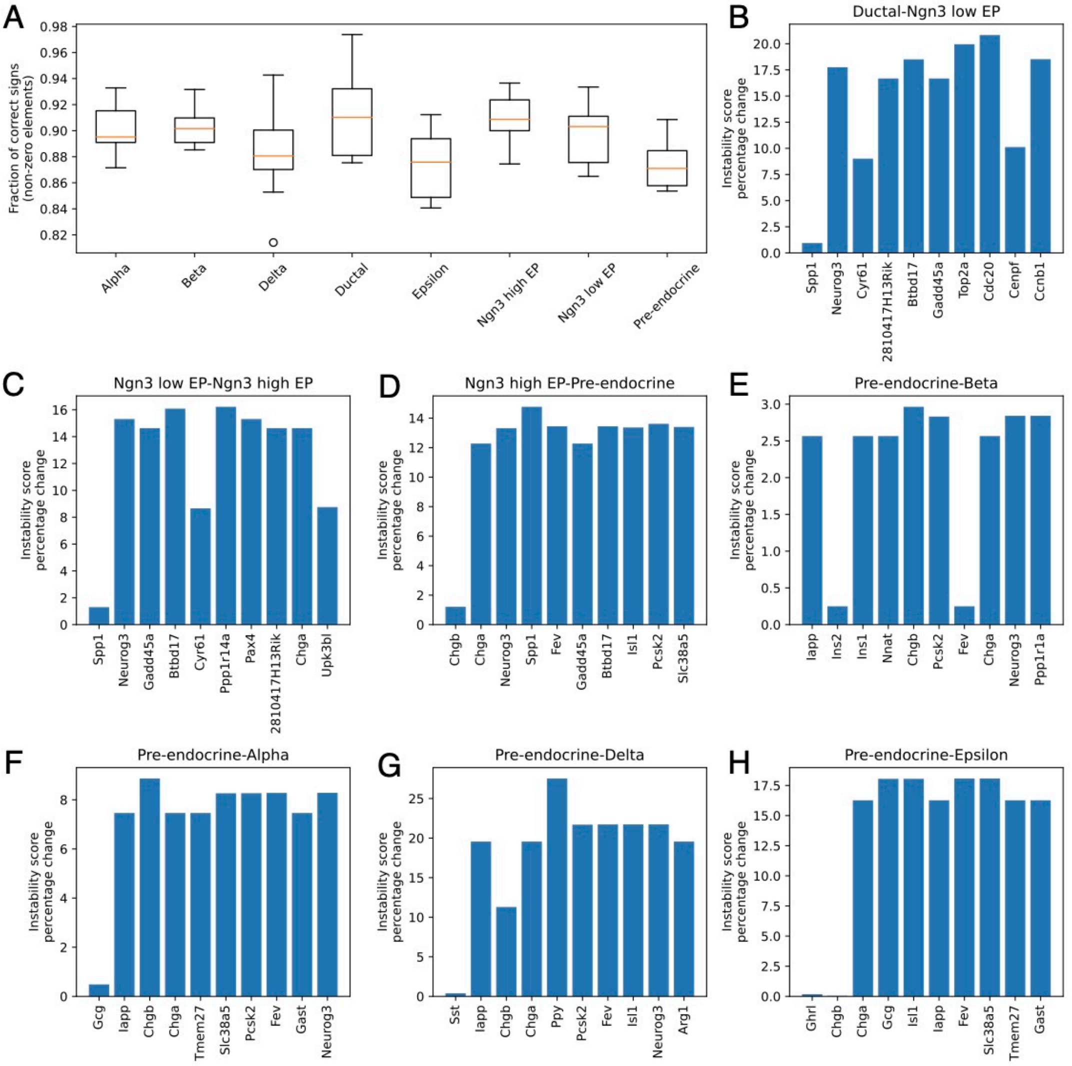

Appendix figure S10. **Pancreas inference robustness upon randomized removal of genes. (A)** The fraction of correct signs captured when comparing the pancreas GRN inference with 50 top genes with a “randomized inference” where 5 of the top genes are chosen and removed randomly. The boxplot highlights the distribution of n=10 independent simulation, each removing 5 different genes chosen randomly. **(B-H)** The percentage change of gene instability score for the 7 transitions in the pancreas dataset when randomly removing 5 of the 50 top expressed genes. Bar plots average the score changes over the n=10 independent simulations.

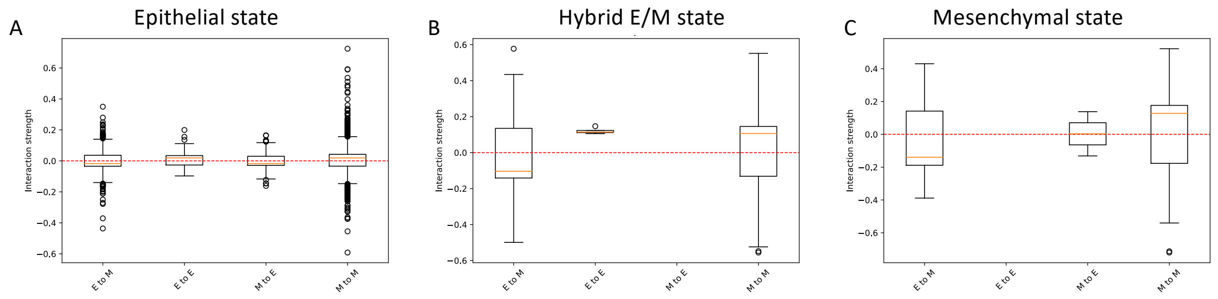

Appendix figure S11. **Distribution of gene-gene interaction coefficients. (A)** Distributions of gene-gene interaction coefficients for epithelial to mesenchymal gene regulation (E to M), epithelial to epithelial genes regulation (E to E), mesenchymal to epithelial genes regulation (M to E), and mesenchymal to mesenchymal genes regulation (M to M) in the gene-gene interaction matrix of the epithelial state. **(B-C)** Same as (A) for the Hybrid E/M and Mesenchymal states, respectively.

Appendix Tables

Method	EMT-epi	EMT-Hyb	EMT-Mes	Cycle	Bif-1	Bif-2	Truf-1	Trif-2	Trif-3
GENIE3	0.23	0.24	0.22	0.26	0.12	0.24	0.30	0.24	0.32
GRISLI	NA*	NA*	NA*	0.21	0.21	0.11	0.32	0.46	0.34
GRNBOOST2	0.22	0.21	0.27	0.25	0.12	0.22	0.30	0.24	0.30
GRNVBEM	0.15	0.18	0.14	0.32	0.21	0.17	0.22	0.34	0.33
LEAP	0.22	0.19	0.22	0.23	0.21	0.46	0.26	0.28	0.28
PIDC	0.20	0.15	0.13	0.30	0.12	0.28	0.30	0.2	0.34
PPCOR	0.18	0.14	0.17	0.26	0.13	0.15	0.30	0.24	0.32
SCNS	0.21	0.26	0.17	0.35	0.15	0.16	0.25	0.25	0.27
SCODE	NA	NA	NA	0.16	0.13	0.15	0.36	0.25	0.34
SCRIBE	0.15	0.24	0.11	0.20	0.14	0.14	NA	0.25	0.24
SINCERITIES	0.19	0.25	0.12	0.18	0.18	0.21	0.26	0.30	0.31
SINGE	NA	NA	NA	0.16	0.15	0.13	0.26	0.30	0.29
spliceJAC	0.20	0.34	0.35	1.00	0.31	0.54	0.57	0.61	0.52

Appendix Table S1. AUPRC scores for spliceJAC and existing GRN inference methods, rounded to the second decimal units. NA=no output file was generated. NA*=an output file without any predicted edge was generated. Green highlights the method with highest AUPRC for each combination of circuit/state All scores and source code for GRN inference benchmarking are available at: https://github.com/cliffzhou92/jacobian-inference-benchmarking.
