# Supplementary material for: spliceJAC: transition genes and state‐specific gene regulation from single‐cell transcriptome data
Source: Mol Syst Biol. 2022 Nov 2;18(11):e11176. doi: 10.15252/msb.202211176 (PMC9627675; doi:10.15252/msb.202211176)
Supplement: Supplementary file 2 — Expanded View Figures PDF [file MSB-18-e11176-s001.pdf]

Expanded View Figures

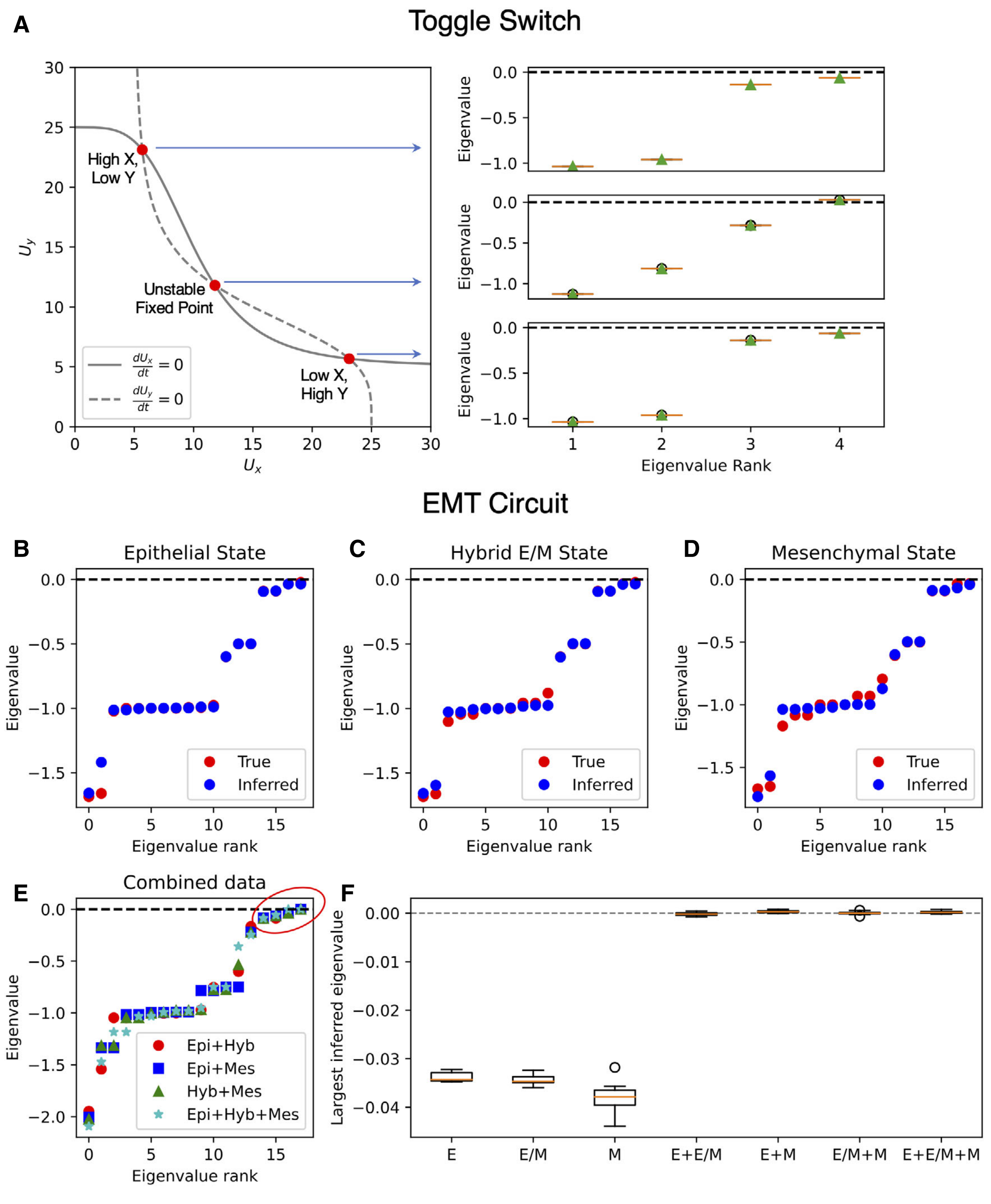

Figure EV1. Spectral analysis of unstable fixed points.

A Left: The phase space of the toggle switch synthetic circuit. Right: The eigenvalues of the Jacobian matrices in the three fixed points, ranked in ascending order, inferred by spliceJAC.

B–D The ground truth (red) and inferred (blue) eigenvalue spectrum in the three fixed points of the EMT circuit.

E The inferred eigen-spectrums cells sampled from multiple cell states are merged. Positive eigenvalues in the red circle indicate instability.

F Comparison of largest eigenvector inferred by spliceJAC in the three stable states and in the cell mixtures. The boxplot central bands and boxes depict average, first to third quantile (Q1–Q3) range, respectively, while the whisker extension corresponds to 1.5× the interquartile range (IQR). The boxplot results are computed over 10 independent simulations.

 ©2022 The Authors

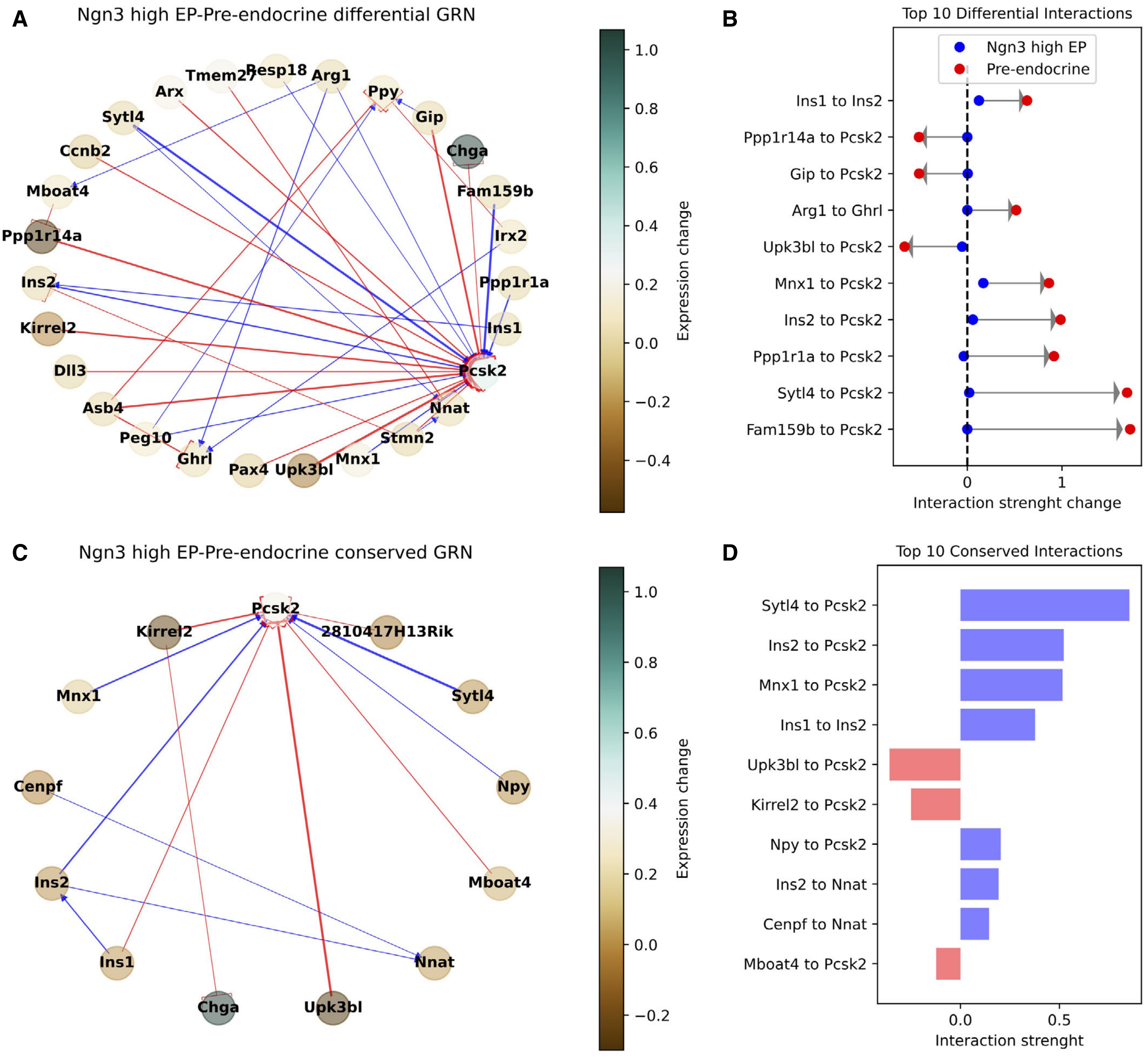

Figure EV2. The differential and conserved interactions in the Ngn3 high EP/Pre-endocrine cell state transition.

A The differential GRN between the Ngn3 high EP and Pre-endocrine cell states. Node colormap indicates the gene expression fold-change between the cell states.

B The top 10 differential interactions between the Ngn3 high EP and Pre-endocrine cell states. Arrows depict the interaction strength change.

C, D The conserved GRN (C) and top 10 conserved interactions (D) highlight the interactions that maintained similar strength from the Ngn3 high EP to the Pre-endocrine cell state.

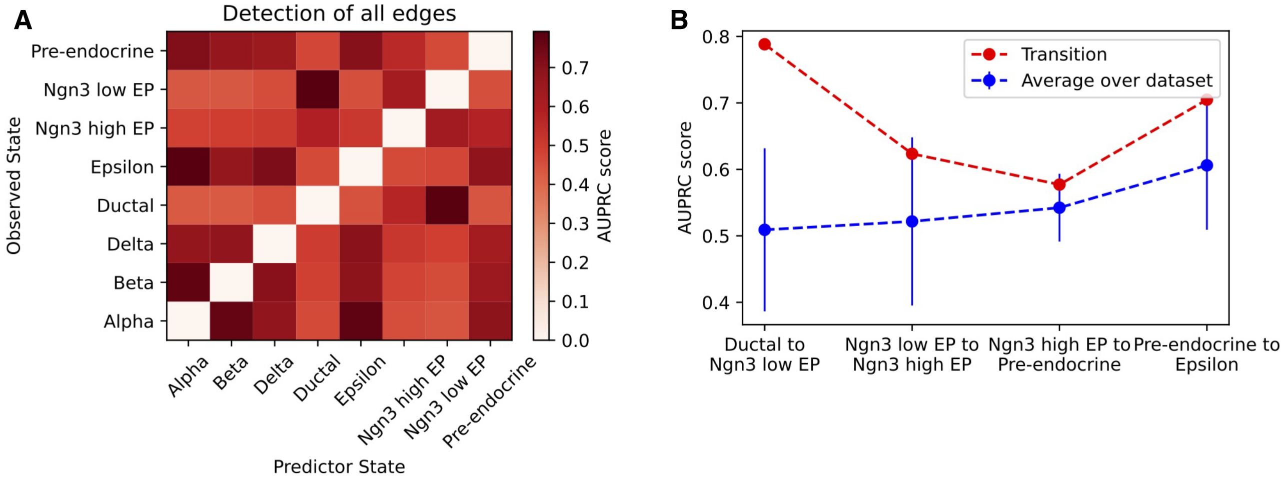

Figure EV3. The GRN similarity along a developmental trajectory.

A The area under the precision-recall curve (AUPRC) when predicting the GRN of the observed GRN (*y*-axis) using the GRN of the predictor state (*x*-axis).

B The goodness of GRN prediction along a developmental trajectory. For each point, the red point shows the AUPRC between the GRNs of the starting and final state. For comparison, the blue dots and error bars show the average and standard deviation (SD) of AUPRC obtained when comparing the GRN of the starting state to the GRN of any other state in the dataset (n = 6 other states in the dataset excluding the final state).

 ©2022 The Authors
