# Supplementary material for: spliceJAC: transition genes and state‐specific gene regulation from single‐cell transcriptome data
Source: Mol Syst Biol. 2022 Nov 2;18(11):e11176. doi: 10.15252/msb.202211176 (PMC9627675; doi:10.15252/msb.202211176)
Supplement: Supplementary file 4 [file MSB-18-e11176-s002.pdf]

spliceJAC: Transition genes and state specific gene regulation from single-cell transcriptome data

Qing Nie, Peijie Zhou, and Federico Bocci
DOI: 10.15252/msb.202211176

Corresponding author(s): Qing Nie (qnie@math.uci.edu) , Peijie Zhou (peijiez1@uci.edu)

Review Timeline:

Submission Date:	8th Jun 22
Editorial Decision:	26th Jul 22
Revision Received:	14th Sep 22
Editorial Decision:	6th Oct 22
Revision Received:	7th Oct 22
Accepted:	10th Oct 22

Editor: Jingyi Hou

Transaction Report:

(Note: With the exception of the correction of typographical or spelling errors that could be a source of ambiguity, letters and reports are not edited. Depending on transfer agreements, referee reports obtained elsewhere may or may not be included in this compilation. Referee reports are anonymous unless the Referee chooses to sign their reports.)

Thank you for submitting your work to Molecular Systems Biology. We have now heard back from two of the three reviewers who agreed to evaluate your manuscript. Unfortunately, after a series of reminders, we did not manage to obtain a report from Reviewer #2. In the interest of time, and since the recommendations of Reviewers #1 and #3 are quite similar, we have decided to proceed with these two reports. As you will see below, the reviewers acknowledge the novelty and potential interest of the study. They raise, however, a series of concerns, which we would ask you to address in a major revision.

I think the reviewers' recommendations are relatively clear, so there is no need to reiterate the points listed below. All issues raised by the reviewers need to be satisfactorily addressed. As you may already know, our editorial policy allows in principle a single round of major revision, and it is therefore essential to provide responses to the reviewers' comments that are as complete as possible.

On a more editorial level, we would ask you to address the following issues:

Reviewer #1:

The manuscript presents spliceJAC, a novel approach for construction of Gene Regulatory Networks, using the expansion of splicing kinetics dynamics around stable cell states. The approach is theoretically very interesting and visionary, well written and has a high potential for further development and generalisations in the field for application scRNA-seq data sets. Here are my questions to the authors:

- The authors mention the criterion of no. genes > no. cells for the method to be able to construct the GRN, however in the shown example the number of genes included is not more than a dozen whereas the number of cells is typically huge in scRNA-seq experiments. There seems to be other limiting factors more decisive than the number of cells which needs clarification.

- Given a list of genes and data near a stable cell state, is the GRN inferred by spliceJAC a unique answer?

- In any case, a selected list of genes is very likely missing some of the important genes that also play a regulatory role in cell state transitions. How would missing a few such genes affect the inference of interaction among other genes, and the downstream analysis results? Can you show with a simulation?

- In general a given GRN can have more than one attractor states. Is it always necessary for spliceJAC to associate every stable cell state to a different GRN?

- One would expect the regulatory circuitry of two adjacent stable cell states to be not too different from each other. Does spliceJAC indicate any such similarities between the inferred GRNs? There are also other reasons for comparing the GRN of two cell states. However, it is super difficult to compare the GRNs by the current visualisation (e.g. in Fig S2A), as the graph visualisations seem to be produced completely independently for each cell state. Could the authors use fixed positions for the same genes that appear in multiple GRNs or facilitate graph comparisons in other ways?

- Generally, an stable state is a local energy minimum, thus is an attractor from many directions, i.e. small perturbations in any direction would converge back to the attractor basin. This is also the case for the simulation presented in Fig. 1A. However, in cell differentiation cells flow to the (meta)stable state from one side but exit it from another side, as cell differentiation is a directed process. Doesn't this disturb any of the assumptions of the mathematical model for doing a linear expansion near the stable state?

- In Fig. S3 D-F the authors write "The Jacobian matrices are compared to the "correct" Jacobian (inferred following the procedure presented in Methods, section 1)". Does this mean the inferred matrix using the maximum number of cells? If so are there also any other methods used for validation of GRN, e.g. interactions reported already in data bases or literature?

- How far is spliceJAC from a reliable GRN construction and comparison tool that biologists would widely use and get new biological insight into their data? What are the cleanest and best scenarios for application in its current state?

Reviewer #3:

Summary

The authors developed a new computational method, spliceJAC, to use both spliced and unspliced mRNA from scRNAseq data to infer GRN and identify putative drive genes between stable cell states. spliceJAC assumes cell annotations correspond to stable cell states and provide validation for such assumption. The authors benchmarked the utilities of the tool against six other GRN construction tools with two synthetic datasets, one pancreas dataset, and one lung tumor dataset.

Major comments

1. The synthetic dataset used for benchmarking spliceJAC against other GRN inference algorithms is overly simplistic. To make a more robust comparison, please include more diverse and complex datasets from the Beeline pipeline as well as their benchmarking statistics. The cycling and the bifurcating converging datasets from the Beeline pipeline will be particularly interesting to test out how spliceJAC handles non-unidirectional transitions.
2. The authors mentioned in the text that the downstream stability analysis can provide the self-consistent validation for such assumption that there are stable cell states assumed by the cluster annotation. It would be crucial to include a scenario/test case where the cell states are not stable, but the downstream instability score can help to evaluate the assumption.
3. Since the authors are using the RNAvelocity framework to compute for the spliced and unspliced mRNA, when identifying key driver genes between the cell states (e.g. Fig 3), it would be interesting to compare what are the driver genes that can be identified by RNAvelocity or just simple differential gene expression analysis, and what can only uniquely be identified by spliceJAC.

Minor comments

1. The sparsely documented Github page (as well as the example jupyter notebook) will strongly hinder the usage of spliceJAC in the community. There is also no available code to examine the reproducibility of the analysis.
2. It is unclear if the authors' definition of cell state is different than cell types.
3. It is also unclear what biological insights were gained from Figure 3H and Figure 4J. Why was it interesting that TGs play an important role in organelle and extracellular region processes whereas the complete EMT genes are more highly represented in the endomembrane system?

Response to Reviewer #1

Comment: *The manuscript presents spliceJAC, a novel approach for construction of Gene Regulatory Networks, using the expansion of splicing kinetics dynamics around stable cell states. The approach is theoretically very interesting and visionary, well written and has a high potential for further development and generalisations in the field for application scRNA-seq data sets.*

Response: We are glad that the reviewer finds our approach "visionary" and that the manuscript is "well-written and has high potential". We also would like to thank the reviewer for their comments which really improved the content and presentation of the manuscript. We have included below detailed answers for the specific comments.

Comment: *Here are my questions to the authors:*

- The authors mention the criterion of no. genes > no. cells for the method to be able to construct the GRN, however in the shown example the number of genes included is not more than a dozen whereas the number of cells is typically huge in scRNA-seq experiments. There seems to be other limiting factors more decisive than the number of cells which needs clarification.

Response: The reviewer raises a great point about spliceJAC's capability to deal with large numbers of genes. The limiting factor is that spliceJAC solves the gene-gene interaction model one cluster at a time. Moreover, to compare the genes' role across clusters, we aim at selecting the same set of genes for all clusters. Invariably, rarer cell populations represented by fewer cells will always set the maximum allowed number of genes. Additionally, our plotting tool includes a controllable parameter to select only the top percentage of key interactions when plotting the predicted GRN, thus resulting in smaller GRNs with less nodes. While this feature allows easier visualization of smaller networks, we acknowledge a lack of explanation. In the revised manuscript, we have further explained and provided examples.

First, in the "**Overview of spliceJAC**" section, we previously explained that "*The number of selected key interactions and size of the network can be controlled by the user based on a quantile selection feature*". Furthermore, when discussing the pancreas endocrinogenesis data, we observe that "*Delta represents the rarest population, with only 70 identified cells, thus putting an upper limit to the number of genes considered in the spliceJAC inference*". We have also explained in the Methods and Protocols section 8 "**Analysis of the pancreas epithelium dataset**", where we added the sentence: "*The size of the Delta cell population (n=70 cells) defines the upper limit for the number of selected genes for spliceJAC inference. Moreover, since spliceJAC employs a bootstrapping method where the gene-gene interaction inference is repeated multiple times (nsim=10 by default) using only a fraction p of cells (p=0.9 by default), the inference is limited to a theoretical maximum of* $p\,n = 63$ *genes. Therefore, the 50 top highly expressed genes were selected*".

Moreover, we have improved the supplementary figure (***Appendix S5***) with an intuitive visualization of how the GRN of the Ductal cell state scales based on the fraction of top interactions selected. In the main text, we write that "*The gene regulatory networks*

associated to each cell state can be visualized through heatmaps or network representation with varying levels of detail based on quantile weight selection feature (Appendix S5B-E)".

Comment: *Given a list of genes and data near a stable cell state, is the GRN inferred by spliceJAC a unique answer?*

Response: spliceJAC employs linear, ridge, or lasso regression to infer state-specific interaction matrices. Therefore, there is a guaranteed unique solution if the number of observables (cells in the cell state) is larger than the number of variables (the gene-gene interaction coefficients). This motivates our choice to set an upper bound on the size of the interaction matrix based on the size of the smallest cell population (as also discussed in the previous question). If the number of selected genes exceeds the number of observed cells, a unique solution cannot be guaranteed.

To be clear on this point, in the revision we have included an explanation in the Result section titled "**Modeling stability and cell state specific regulation with multivariate mRNA splicing analysis**": "*To ensure a unique solution to the regression problem, the number of genes cannot exceed the number of observations (i.e., cells). Therefore, the size of the number of cells within a given cell state defines as an upper limit to the number of inferred interactions*". We have also expanded the explanation in the **Methods and Protocol section 1** with the comment: "*Therefore, at least N independent observations (i.e., cells) are required within each cell state to guarantee a unique solution to the regression problem*".

Comment: *In any case, a selected list of genes is very likely missing some of the important genes that also play a regulatory role in cell state transitions. How would missing a few such genes affect the inference of interaction among other genes, and the downstream analysis results? Can you show with a simulation?*

Response: The reviewer touches on a key point of the method, since there will likely be important genes that are missing in many datasets. To address this point systematically, in the revision, we have quantified the effect of missing genes on GRN and transition gene inference in both a synthetic and experimental dataset.

First, we have tested the effect of removing one gene at a time in the synthetic tristable EMT circuit, which has a total of 9 genes. First, we generated the synthetic data with all the genes, and later deleted the counts for one gene at a time from the spliceJAC input before running the regression. In the new **Appendix figure S2**, we visually show the inferred gene-gene interactions for the Mesenchymal state, demonstrating that spliceJAC still captures the remaining interactions well. Moreover, we compared the quality of the inference using the area under the receiving-operating characteristic (AUROC) and precision recall curve (AUPRC) for all three states, showing that the inference is robust. In the main text section "**spliceJAC reconstructs state-specific gene-gene interactions from in silico circuits**", we comment that "*Moreover, the inference was relatively robust even when genes were removed, one at a time, from the spliceJAC count matrix input, and the inference was run on the remaining subset of genes (Appendix Figure S2). This approach confirms the inference robustness in silico when missing genes might play important regulatory roles.*".

Moreover, we have tested the effect of gene removal in the pancreas endocrinogenesis dataset (**Appendix figure S10**). For this case, we considered our inference results with the top 50 genes as the "ground truth". Then, we run 10 independent simulations where we randomly removed 5 of the top 50 genes. spliceJAC is still able to capture about 90% of correct signs when considering the non-zero elements of the GRN adjacency matrices (i.e., the pairs of genes that interact). Moreover, we have quantified the percentage change of the transition genes instability scores. For each transition in the pancreas dataset, the instability scores changed by at most 15-20%, and many genes exhibit much lower percentage changes of 2-3%. Overall, these results demonstrate that spliceJAC can still capture interaction signs and predict transition genes with high fidelity even in situations where important genes are omitted. In the main text section "**Identification of cell state-specific regulatory interactions in the pancreas epithelium**", we comment that "*The consistency of GRN inference and transition gene analysis was further confirmed when randomly removing subsets of genes from the dataset, which mimics missing genes that can either be undetected in the dataset or not selected for the spliceJAC analysis (Appendix Figure S10)*".

Comment: *In general a given GRN can have more than one attractor states. Is it always necessary for spliceJAC to associate every stable cell state to a different GRN?*

Response: In spliceJAC, we propose to linearly expand the gene-gene interactions around each stable cell state, and thus obtaining linearized, state-specific GRNs. The state-specific expansion is linear, and therefore has a unique solution. An intuitive example of this logic is the synthetic EMT circuit that gives rise to 3 stable cell states (presented in Figure 2E-F). While this system is defined by a unique, multistable circuit, each cell state is characterized by a different Jacobian matrix, which is the linear expansion of the circuit around the cell state specifically. The analysis provided by spliceJAC is valuable because different edges of the GRN are active in the different cell states, a layer of description that eludes "global" GRN methods that disregard cell states.

In the revision we have further clarified this point in the "**Modeling stability and cell state specific regulation with multivariate mRNA splicing analysis**" section: "*Therefore, while eqs. (3) can describe a nonlinear regulatory circuit with potentially multiple stable states, we linearize the circuit interactions around each stable cell states that are thus described by different parameters*".

Comment: *One would expect the regulatory circuitry of two adjacent stable cell states to be not too different from each other. Does spliceJAC indicate any such similarities between the inferred GRNs? There are also other reasons for comparing the GRN of two cell states. However, it is super difficult to compare the GRNs by the current visualisation (e.g. in Fig S2A), as the graph visualisations seem to be produced completely independently for each cell state. Could the authors use fixed positions for the same genes that appear in multiple GRNs or facilitate graph comparisons in other ways?*

Response: We thank the reviewer for this interesting suggestion. In the revised manuscript, we have introduced many metrics and visualization tools to better compare GRNs.

First, we have included plotting tools to visualize the "differential" and "conserved" GRNs when comparing two cell states. These networks are defined based on the GRN edges with

smallest and largest changes between the two states. An explanation of this analysis has been added in the **Methods and Protocol section 4**. These GRN visualizations are also supported by new analysis tools to visualize the top differential and top conserved interactions in details. Furthermore, these plotting functions are quite flexible and enable users to compare any pair of cell states and inspect a controllable number of top differential or conserved interactions. We applied these new tools to identify the top conserved and differential interactions in the transition from Ngn3 High EP to Pre-endocrine cell state in the pancreas dataset, and the results are shown in the **Expanded view figure 2 (EV2)**. In the main text section "**Identification of cell state-specific regulatory interactions in the pancreas epithelium**", we comment that "*The implications of cell state transition on gene regulation can be captured by differential and conserved GRNs that rank the top gene-gene interactions with largest or smallest change between the starting and final states (Figure EV2).*".

Furthermore, we have introduced more quantitative comparison tools to quantify the change of GRN between cell states based on the area under the receiver-operator characteristic or precision-recall curve (AUROC/AUPRC). These metrics test whether the GRN of the "starting" state of a transition is a good predictor for the GRN of the "final" state. In the newly introduced **Expanded view figure 3 (EV3)**, we picked a differentiation trajectory through multiple cell states (Ductal-Ngn3 low EP- Ngn3 high EP-Pre endocrine-Epsilon) and compared AUPRC scores of consecutive cell states against AUPRC scores of pairs of cell states picked randomly. As the reviewer correctly suggested, GRNs of adjacent cell states were shown to be "closer" (i.e., higher AUPRC scores) compared to GRNs of faraway cell states. In the main text section "**Identification of cell state-specific regulatory interactions in the pancreas epithelium**", we write that "*It may be expected that adjacent cell states share more similarities in GRN structure compared to the states that are not directly connected by a transition. Comparing the GRNs along a developmental trajectory from the Ductal to the Alpha state shows that the GRNs of adjacent cell states Along the transition retain more similarity compared to GRNs of non-adjacent cell states, as quantified by the area under the precision-recall curve (AUPRC). In other words, the GRN of the "starting states" are typically good predictors for the GRNs of the "final states" (Figure EV3)*".

Overall, these newly added functionalities greatly improved the potential to investigate and compare GRNs for spliceJAC's users.

Comment: *Generally, an stable state is a local energy minimum, thus is an attractor from many directions, i.e. small perturbations in any direction would converge back to the attractor basin. This is also the case for the simulation presented in Fig. 1A. However, in cell differentiation cells flow to the (meta)stable state from one side but exit it from another side, as cell differentiation is a directed process. Doesn't this disturb any of the assumptions of the mathematical model for doing a linear expansion near the stable state?*

Response: The reviewer correctly points out that directionality is a key aspect of cell state transitions. When calculating the gene instability scores, spliceJAC first computes the more unstable directions (or unstable manifold) of the starting cell state, which do not consider the specific direction of transition. In the second step of the modeling, however, the unstable manifold is projected onto the path connecting starting and final states, thus selecting unstable directions that are aligned to the specific transition path. In the revision, we have clarified this modeling assumption in the **Methods and Protocols section 5** by noting that "*The unstable manifold projection onto the transition path ensures that, even though unstable*

eigenvectors might point in different directions in the gene space, the directionality of cell state transition is preserved". Furthermore, we have clarified this aspect in the result section "**Overview of spliceJAC**". The revised explanation now reads: "*To identify important transition driver genes, spliceJAC projects the starting state's unstable manifold onto the path connecting starting and target cell states, thus considering the specific transition direction in the gene landscape*."

Comment: *In Fig. S3 D-F the authors write "The Jacobian matrices are compared to the "correct" Jacobian (inferred following the procedure presented in Methods, section 1)". Does this mean the inferred matrix using the maximum number of cells? If so are there also any other methods used for validation of GRN, e.g. interactions reported already in data bases or literature?*

Response: We apologize for the confusion. As the reviewer correctly points out, we refer to the inferred Jacobian matrix when all cells in the state are considered. In the revision we have modified the caption of **Appendix figure S7** to clearly state: "*The Jacobian matrices inferred with cell subsampling are compared to the reference state-specific Jacobian matrices inferred with the standard spliceJAC pipeline*".

However, the reviewer hints at another complex problem: how to properly benchmark the predicted GRNs using existing information. First, in the revised version, we have introduced more quantitative benchmarking against multiple synthetic circuits including limit cycle, bifurcating converging, and trifurcating circuits, using the Beeline simulation and benchmarking pipelines. In the revised **Figure 2K** and **Appendix Table S1**, we show that spliceJAC outperforms existing GRN inference methods when focusing on cell state-specific interactions. Together, these tests demonstrate the robustness when spliceJAC is applied to different circuit topology and behaviors. The commands and raw data generated are available in the repository: https://github.com/cliffzhou92/jacobian-inference-benchmarking.

Moreover, in the revision we have improved the **Discussion** to propose how to incorporate prior biological knowledge in the future: "*incorporating additional layers of datasets such as chromatin accessibility or proteomics could significantly enhance the accuracy of gene-interaction terms. Further, prior knowledge of gene interactions may help to reduce the false positives of regulation recovery. In the future, these additional measurements can be incorporated with spliceJAC to generate more data-driven and robust predictions*".

Comment: *How far is spliceJAC from a reliable GRN construction and comparison tool that biologists would widely use and get new biological insight into their data? What are the cleanest and best scenarios for application in its current state?*

Response: We thank the reviewer for encouraging us in sharpening our message. The results presented in the manuscript and response to the questions raised by the reviewers demonstrate spliceJAC's ability to provide novel biological insight, which could be further refined in the future by introducing prior knowledge in the GRN inference. Furthermore, we have optimized and improved the documentation of the package to make it readily usable by biologists and bioinformaticians. We have summarized the best cases for application for the spliceJAC model and package in the **Discussion** section: "*Together, spliceJAC has three important functions that allow novel biological insights: (1) Distinguishing transition driver*

genes leading to different final states that stem from a common initial state, such as the differentiation of Pre-endocrine cells into either Alpha, Beta, Delta and Epsilon cells in during pancreas endocrinogenesis; (2) Characterizing gene regulation in intermediate cell states that are not transitory but rather act as stable attractors, such as the hybrid Epithelial/Mesenchymal state during EMT; and (3) Quantifying the differences in gene signaling roles between distinct cell states".

Response to Reviewer #3

Summary *The authors developed a new computational method, spliceJAC, to use both spliced and unspliced mRNA from scRNAseq data to infer GRN and identify putative drive genes between stable cell states. spliceJAC assumes cell annotations correspond to stable cell states and provide validation for such assumption. The authors benchmarked the utilities of the tool against six other GRN construction tools with two synthetic datasets, one pancreas dataset, and one lung tumor dataset.*

Response: We thank the reviewer for the constructive comments that greatly improved the manuscript. Please find below a detailed point-by-point response.

Major comments

1. The synthetic dataset used for benchmarking spliceJAC against other GRN inference algorithms is overly simplistic. To make a more robust comparison, please include more diverse and complex datasets from the Beeline pipeline as well as their benchmarking statistics. The cycling and the bifurcating converging datasets from the Beeline pipeline will be particularly interesting to test out how spliceJAC handles non-unidirectional transitions.

Response: We thank the reviewer for suggesting additional strategies to test spliceJAC. In the revised manuscript, we have applied the benchmarking statistics employed in the Beeline pipeline, including area under the receiving-operating characteristic (AUROC) and precision recall curve (AUPRC), and used the Beeline published simulation tool (BoolODE) to test the cycling, bifurcating converging and trifurcating synthetic circuits (details on simulation and inference strategies are included in the **Methods and Protocol sections 6** and **Appendix Figure S3**). The generation of the new synthetic data and calculation of AUROC/AUPRC scores were performed using Beeline's code and original parameters to guarantee a fair and unbiased comparison. In the main text section "**spliceJAC reconstructs state-specific gene-gene interactions from in silico circuits**", we explain: "*To test diverse biological scenarios, we further used the BoolODE simulation tool to generate synthetic data for three cases: a cycling circuit exhibiting a limit cycle, a bifurcating converging circuit with two stable states, and a trifurcating circuit with three stable states (Appendix Figure S3) previously studied in the Beeline GRN inference benchmarking package (Methods and Protocols, section 6)*".

In the revision in total we now consider four complex circuits (EMT, cycle, bifurcating and trifurcating) for a total of nine stable states/limit cycles. In the case of the EMT circuit, ground truth Jacobian matrices could be easily calculated. For the three Beeline circuits, a Jacobian could not be calculated due to the Boolean nature of the simulation strategy. For these circuits, we used the input network topologies as ground truth for algorithm benchmarking. The details for benchmarking are provided in the **Methods and Protocols section 7** titled "**Benchmarking and comparison with existing GRN inference methods**".

The new **Figure panel 2K** provides a summary of performance using the AUPRC score for all considered states and circuits. Overall, spliceJAC gained higher scores for all states/circuits. The three states of the EMT circuits were the only instances where some of the other methods occasionally gained comparable scores. However, we further show that spliceJAC is a better predictor for the EMT circuit specifically based on the percentage of

correct interaction signs in the GRN (i.e., positive and negative edges corresponding to activation or inhibition), a feature that most of the other considered algorithms could not capture (showed in Figure 2I-J). In the main text section "**spliceJAC reconstructs state-specific gene-gene interactions from in silico circuits**", we add that "*To quantify the goodness of state specific GRN inference, we used the Beeline package to evaluate the area under the precision recall curve (AUPRC, Methods and Protocols, section 7), showing that spliceJAC consistently achieved better scores for all the simulated synthetic circuits (Figure 2K, Appendix Table S1)*".

Very interestingly, spliceJAC infers very well the cycling circuit, achieving AUROC and AUPRC scores of 1. In the new **Appendix Figure S4** we provide a visual comparison for the cycling circuit to demonstrate the goodness of spliceJAC's inference, especially in terms of distinguish between activation and inhibition. In the main text section "**spliceJAC reconstructs state-specific gene-gene interactions from in silico circuits**", we have commented: "*While the AUPRC calculation does not penalize incorrect sign detection, the inference of the limit cycle highlights spliceJAC's ability to correctly predict not only the circuit topology but the correct signs for activation or inhibition (Appendix Figure S4)*".

To guarantee a transparent and accessible evaluation of our benchmarking, we have uploaded all simulated data and benchmarking results in a public github folder (https://github.com/cliffzhou92/jacobian-inference-benchmarking) that includes instructions to reproduce the data.

2. The authors mentioned in the text that the downstream stability analysis can provide the self-consistent validation for such assumption that there are stable cell states assumed by the cluster annotation. It would be crucial to include a scenario/test case where the cell states are not stable, but the downstream instability score can help to evaluate the assumption.

Response: We agree with the reviewer that this aspect should be further motivated and explained. In the revised manuscript, we have explored different scenarios with the synthetic circuits to test our method's ability to discern between stable and unstable cell states. This analysis is summarized in the **Expanded View figure 1 (EV1)**. In the main text section "**spliceJAC reconstructs state-specific gene-gene interactions from in silico circuits**", we comment that "*To test spliceJAC's ability to discern between stable and unstable cell states, we simulated short trajectories around the unstable fixed point of the toggle switch synthetic circuit, where spliceJAC correctly predicts a positive eigenvalue that is indicative of instability (Figure EV1A). Furthermore, to "simulate" an erroneous mixing of states that could happen in real datasets, we ran spliceJAC on mixtures of cells belonging to multiple cell states of the EMT circuit. In this case, spliceJAC always predicted the largest eigenvalue to be either positive or extremely close to zero (Figure EV1B-F). Overall, these simulations demonstrate spliceJAC's ability to distinguish between stable and unstable states from simulation of synthetic circuits*".

3. Since the authors are using the RNAvelocity framework to compute for the spliced and unspliced mRNA, when identifying key driver genes between the cell states (e.g. Fig 3), it would be interesting to compare what are the driver genes that can be identified by RNAvelocity or just simple differential gene expression analysis, and what can only uniquely be identified by spliceJAC.

Response: The reviewer correctly stresses a key aspect of the spliceJAC method. In the original manuscript, we only included information about differentially expressed genes (DEGs) computed with Scanpy's differential expression function. This was shown, for instance, in the Figure 3E, with a core GRN including the top DEG of the Ngn3 high EP state and the top transition genes identified by spliceJAC. In the revised manuscript, we have included a new analysis shown in the new **Appendix Figure 8B** to compare the instability score predicted by spliceJAC with the "top likelihood genes" computed with scVelo based on their mRNA splicing model for the different transitions in the pancreas endocrinogenesis dataset. The new analysis shows a partial overlap between certain genes, but also a set of putative transition genes that are uniquely identified by spliceJAC. In the revised main text section "**Identification of cell state-specific regulatory interactions in the pancreas epithelium**", we comment that "*We further compared spliceJAC's instability scores with the cluster specific top-likelihood genes identified by scVelo's dynamical modeling, again confirming that many genes are uniquely predicted by spliceJAC (Appendix Figure S8B)*". To summarize, the new version of the Appendix Figure 8 showcases the comparison of spliceJAC's instability scores with DEG scores (Appendix Figure S8A, already existing) and scVelo likelihood scores (Appendix Figure S8B, introduced in the revision).

Minor comments

1. The sparsely documented Github page (as well as the example jupyter notebook) will strongly hinder the usage of spliceJAC in the community. There is also no available code to examine the reproducibility of the analysis.

Response: We agree with the reviewer that the documentation on the method needs to be improved. In the revised version, we have organized spliceJAC into a python library that can be easily installed. Furthermore, we have greatly improved the documentation and provided well-explained notebooks that walk users through the datasets studied in the manuscript.

-The Github repository of the spliceJAC package is accessible at: https://github.com/federicobocci/spliceJAC. This repository also contains the pancreas dataset and source code to generate all results presented in Figure 3 and related supplementary.

-The Github repository with the benchmarking data is accessible at: https://github.com/cliffzhou92/jacobian-inference-benchmarking, with instructions on how simulations and benchmarking were conducted.

2. It is unclear if the authors' definition of cell state is different than cell types.

Response: We thank the reviewer for pointing out this important distinction. By default, spliceJAC takes the cell annotations in the input dataset and treat each distinct annotation as a different cell state. In this sense, cell states and types are considered as synonyms. To an extent, we have left the relation between cell state and type intentionally open to interpretation because we expect that spliceJAC could be applied by the community to very different biological systems with different degrees of prior knowledge about the possible cell phenotypes. We have added a comment in **Discussion** section "*In the present work, the terms "cell state" and "cell type" are largely considered as synonymous. While the term "cell*

state" has a clear relation with well-defined mathematical concepts (such as attractors and stability), the term "cell type" is more loosely defined, and types are usually associated with expression of well-known markers. spliceJAC users can adjust their analysis by modifying the cell annotations input to spliceJAC, for instance by merging or excluding certain cell states based on the prior knowledge on the specific biological system".

3. It is also unclear what biological insights were gained from Figure 3H and Figure 4J. Why was it interesting that TGs play an important role in organelle and extracellular region processes whereas the complete EMT genes are more highly represented in the endomembrane system?

Response: We apologize for the lack of explanation about the gene ontology analysis. This analysis is primarily presented as an example of how spliceJAC's predictions can be used for further downstream analysis in specific biological scenarios. In the revision we have added comments in the main text about the potential implications of these findings in the pancreas endocrinogenesis (Figure 3H): "*This analysis highlights the involvement in distinct biological processes of genes that drive transitions toward different terminal states. For example, the genes associated to the Beta state are well represented in extracellular space regulation, whereas Delta genes are more represented in the intracellular region process*". We have similarly commented about the EMT GO analysis (Figure 4J), where most GO biological processes are equally represented by partial and complete EMT transition genes: "*Overall, most biological processes in the GO analysis exhibit similar representation for partial and complete EMT transition genes, perhaps unexpectedly when considering that these are two steps of the same trans-differentiation process*".

Thank you for sending us your revised manuscript. We have now heard back from the reviewer who agreed to evaluate your study. As you will see, the reviewer is satisfied with the modifications made and thinks the study is now suitable for publication.

Before we can formally accept your manuscript, we would ask you to address the following issues:

Reviewer #1:

The authors have addressed all my questions. I recommend acceptance.

2nd Revision - Editorial Decision 10th Oct 2022

Thank you again for sending us your revised manuscript. We are now satisfied with the modifications made and I am pleased to inform you that your paper has been accepted for publication.

EMBO Press Author Checklist

Corresponding Author Name: Peijie Zhou, Qing Nie	
Journal Submitted to: Molecular Systems Biology	
Manuscript Number: MSB-2022-11176	

USEFUL LINKS FOR COMPLETING THIS FORM
The EMBO Journal - Author Guidelines
EMBO Reports - Author Guidelines
Molecular Systems Biology - Author Guidelines
EMBO Molecular Medicine - Author Guidelines

Reporting Checklist for Life Science Articles (updated January
This checklist is adapted from Materials Design Analysis Reporting (MDAR) Checklist for Authors. MDAR establishes a minimum set of requirements in transparent reporting in the life sciences (see Statement of Task: 10.31222/osf.io/9sm4x). Please follow the journal's guidelines in preparing your
Please note that a copy of this checklist will be published alongside your article.

Abridged guidelines for figures
1. Data
The data shown in figures should satisfy the following conditions:
- the data were obtained and processed according to the field's best practice and are presented to reflect the results of the experiments in an accurate and unbiased manner.
- ideally, figure panels should include only measurements that are directly comparable to each other and obtained with the same assay.
- plots include clearly labeled error bars for independent experiments and sample sizes. Unless justified, error bars should not be shown for technical
- if n<5, the individual data points from each experiment should be plotted. Any statistical test employed should be justified.
- Source Data should be included to report the data underlying figures according to the guidelines set out in the authorship guidelines on Data

2. Captions
Each figure caption should contain the following information, for each panel where they are relevant:
- a specification of the experimental system investigated (eg cell line, species name).
- the assay(s) and method(s) used to carry out the reported observations and measurements.
- an explicit mention of the biological and chemical entity(ies) that are being measured.
- an explicit mention of the biological and chemical entity(ies) that are altered/varied/perturbed in a controlled manner.
- the exact sample size (n) for each experimental group/condition, given as a number, not a range;
- a description of the sample collection allowing the reader to understand whether the samples represent technical or biological replicates (including how many animals, litters, cultures, etc.).
- a statement of how many times the experiment shown was independently replicated in the laboratory.
- definitions of statistical methods and measures:
  - common tests, such as t-test (please specify whether paired vs. unpaired), simple χ2 tests, Wilcoxon and Mann-Whitney tests, can be unambiguously identified by name only, but more complex techniques should be described in the methods section;
  - are tests one-sided or two-sided?
  - are there adjustments for multiple comparisons?
  - exact statistical test results, e.g., P values = x but not P values < x;
  - definition of 'center values' as median or average;
  - definition of error bars as s.d. or s.e.m.

Please complete ALL of the questions below.
Select "Not Applicable" only when the requested information is not relevant for your study.

Materials

Newly Created Materials	Information included in the manuscript?	In which section is the information available? (Reagents and Tools Table, Materials and Methods, Figures, Data Availability Section)
New materials and reagents need to be available; do any restrictions apply?	Not Applicable	

Antibodies	Information included in the manuscript?	In which section is the information available? (Reagents and Tools Table, Materials and Methods, Figures, Data Availability Section)
For **antibodies** provide the following information:<br>- Commercial antibodies: RRID (if possible) or supplier name, catalogue number and or/clone number<br>- Non-commercial: RRID or citation	Not Applicable	

DNA and RNA sequences	Information included in the manuscript?	In which section is the information available? (Reagents and Tools Table, Materials and Methods, Figures, Data Availability Section)
**Short novel DNA or RNA including primers, probes**: provide the sequences.	Not Applicable	

Cell materials	Information included in the manuscript?	In which section is the information available? (Reagents and Tools Table, Materials and Methods, Figures, Data Availability Section)
**Cell lines:** Provide species information, strain. Provide accession number in repository **OR** supplier name, catalog number, clone number, and/**OR** RRID.	Not Applicable	
**Primary cultures:** Provide species, strain, sex of origin, genetic modification status.	Not Applicable	
Report if the cell lines were recently **authenticated** (e.g., by STR profiling) and tested for mycoplasma contamination.	Not Applicable	

Experimental animals	Information included in the manuscript?	In which section is the information available? (Reagents and Tools Table, Materials and Methods, Figures, Data Availability Section)
**Laboratory animals or Model organisms:** Provide species, strain, sex, age, genetic modification status. Provide accession number in repository **OR** supplier name, catalog number, clone number, **OR** RRID.	Not Applicable	
**Animal observed in or captured from the field:** Provide species, sex, and age where possible**.**	Not Applicable	
Please detail **housing and husbandry conditions**.	Not Applicable	

Plants and microbes	Information included in the manuscript?	In which section is the information available? (Reagents and Tools Table, Materials and Methods, Figures, Data Availability Section)
**Plants:** provide species and strain, ecotype and cultivar where relevant, unique accession number if available, and source (including location for collected wild specimens).	Not Applicable	
**Microbes:** provide species and strain, unique accession number if available, and source.	Not Applicable	

Human research participants	Information included in the manuscript?	In which section is the information available? (Reagents and Tools Table, Materials and Methods, Figures, Data Availability Section)
If collected and within the bounds of privacy constraints report on age, sex and gender or ethnicity for all study participants.	Not Applicable	

Core facilities	Information included in the manuscript?	In which section is the information available? (Reagents and Tools Table, Materials and Methods, Figures, Data Availability Section)
If your work benefited from core facilities, was their service mentioned in the acknowledgments section?	Not Applicable	

Design

Study protocol	Information included in the manuscript?	In which section is the information available? (Reagents and Tools Table, Materials and Methods, Figures, Data Availability Section)
If study protocol has been **pre-registered, provide DOI in the manuscript**. For clinical trials, provide the trial registration number **OR** cite DOI.	Not Applicable	
Report the **clinical trial registration number** (at ClinicalTrials.gov or equivalent), where applicable.	Not Applicable	

Laboratory protocol	Information included in the manuscript?	In which section is the information available? (Reagents and Tools Table, Materials and Methods, Figures, Data Availability Section)
Provide DOI OR other citation details if **external detailed step-by-step protocols** are available.	Not Applicable	

Experimental study design and statistics	Information included in the manuscript?	In which section is the information available? (Reagents and Tools Table, Materials and Methods, Figures, Data Availability Section)
Include a statement about **sample size** estimate even if no statistical methods were used.	Not Applicable	
Were any steps taken to minimize the effects of subjective bias when allocating animals/samples to treatment (e.g. **randomization procedure**)? If yes, have they been described?	Not Applicable	
Include a statement about **blinding** even if no blinding was done.	Not Applicable	
Describe **inclusion/exclusion criteria** if samples or animals were excluded from the analysis. Were the criteria pre-established?<br><br>If sample or data points were omitted from analysis, report if this was due to attrition or intentional exclusion and provide justification.	Not Applicable	
For every figure, are **statistical tests** justified as appropriate? Do the data meet the assumptions of the tests (e.g., normal distribution)? Describe any methods used to assess it. Is there an estimate of variation within each group of data? Is the variance similar between the groups that are being statistically compared?	Not Applicable	

Sample definition and in-laboratory replication	Information included in the manuscript?	In which section is the information available? (Reagents and Tools Table, Materials and Methods, Figures, Data Availability Section)
In the figure legends: state number of times the experiment was **replicated** in laboratory.	Not Applicable	
In the figure legends: define whether data describe **technical or biological replicates**.	Not Applicable	

Ethics

Ethics	Information included in the manuscript?	In which section is the information available? (Reagents and Tools Table, Materials and Methods, Figures, Data Availability Section)
Studies involving **human participants**: State details of **authority granting ethics approval** (IRB or equivalent committee(s), provide reference number for approval.	Not Applicable	
Studies involving **human participants**: Include a statement confirming that **informed consent** was obtained from all subjects and that the experiments conformed to the principles set out in the WMA Declaration of Helsinki and the Department of Health and Human Services Belmont Report.	Not Applicable	
Studies involving **human participants:** For publication of **patient photos**, include a statement confirming that consent to publish was obtained.	Not Applicable	
Studies involving experimental **animals**: State details of **authority granting ethics approva**l (IRB or equivalent committee(s), provide reference number for approval. Include a statement of compliance with ethical regulations.	Not Applicable	
Studies involving **specimen and field samples:** State if relevant **permits** obtained, provide details of authority approving study; if none were required, explain why.	Not Applicable	

Dual Use Research of Concern (DURC)	Information included in the manuscript?	In which section is the information available? (Reagents and Tools Table, Materials and Methods, Figures, Data Availability Section)
Could your study fall under dual use research restrictions? Please check biosecurity documents and list of **select agents and toxins** (CDC): https://www.selectagents.gov/sat/list.htm	Not Applicable	
If you used a select agent, is the security level of the lab appropriate and reported in the manuscript?	Not Applicable	
If a study is subject to dual use research of concern regulations, is the name of the **authority granting approval and reference number** for the regulatory approval provided in the manuscript?	Not Applicable	

Reporting

The MDAR framework recommends adoption of discipline-specific guidelines, established and endorsed through community initiatives. Journals have their own policy about requiring specific guidelines and recommendations to complement MDAR.

Adherence to community standards	Information included in the manuscript?	In which section is the information available? (Reagents and Tools Table, Materials and Methods, Figures, Data Availability Section)
State if relevant guidelines or checklists (e.g., **ICMJE, MIBBI, ARRIVE, PRISMA**) have been followed or provided.	Not Applicable	
For **tumor marker prognostic studies,** we recommend that you follow the **REMARK** reporting guidelines (see link list at top right). See author guidelines, under 'Reporting Guidelines'. Please confirm you have followed these guidelines.	Not Applicable	
For **phase II and III randomized controlled trials**, please refer to the **CONSORT** flow diagram (see link list at top right) and submit the CONSORT checklist (see link list at top right) with your submission. See author guidelines, under 'Reporting Guidelines'. Please confirm you have submitted this list.	Not Applicable	

Data Availability

Data availability	Information included in the manuscript?	In which section is the information available? (Reagents and Tools Table, Materials and Methods, Figures, Data Availability Section)
Have **primary datasets** been deposited according to the journal's guidelines (see 'Data Deposition' section) and the respective accession numbers provided in the Data Availability Section?	Not Applicable	
Were **human clinical and genomic datasets** deposited in a public access-controlled repository in accordance to ethical obligations to the patients and to the applicable consent agreement?	Not Applicable	
Are **computational models** that are central and integral to a study available without restrictions in a machine-readable form? Were the relevant accession numbers or links provided?	Yes	Methods and Protocols, section 11 (Software availability); Data availabiliy section
If publicly available data were reused, provide the respective **data citations in the reference list**.	Yes	Data ref: Bastidas-Ponce et al, 2019; Cook & Vanderhyden, 2020
